# A nondepleting anti-CD19 antibody impairs B cell function and inhibits autoimmune diseases

**DOI:** 10.1172/jci.insight.166137

**Published:** 2023-07-10

**Authors:** Jeffrey S. Boyles, Dorota Sadowski, Scott Potter, Aleksandra Vukojicic, James Parker, William Y. Chang, Yanfei L. Ma, Mark G. Chambers, James Nelson, Barbra Barmettler, Eric M. Smith, Kara Kersjes, Evan R. Himes, Chaohua Lin, Jonathan Lucchesi, Jaladhi Brahmbhatt, Ramtin Sina, Jennifer A. Martin, Evan Maestri, Christopher M. Wiethoff, Gregory L. Dyas, Matthew D. Linnik, Songqing Na, Derrick R. Witcher, Alison Budelsky, Kira Rubtsova

**Affiliations:** 1 Biotechnology Discovery Research, Lilly Research Laboratories, Eli Lilly and Company, Indianapolis, Indiana, USA.; 2 Immunology Discovery, Lilly Biotechnology Center, Lilly Research Laboratories, Eli Lilly and Company, San Diego, California, USA.; 3 Immunology Discovery, Lilly Research Laboratories, Eli Lilly and Company, Indianapolis, Indiana, USA.; 4 Biotechnology Discovery Research, Lilly Biotechnology Center, Lilly Research Laboratories, Eli Lilly and Company, San Diego, California, USA.

**Keywords:** Autoimmunity, Immunology, Adaptive immunity, Immunoglobulins

## Abstract

B cells contribute to multiple aspects of autoimmune disorders, and B cell–targeting therapies, including B cell depletion, have been proven to be efficacious in treatment of multiple autoimmune diseases. However, the development of novel therapies targeting B cells with higher efficacy and a nondepleting mechanism of action is highly desirable. Here we describe a nondepleting, high-affinity anti–human CD19 antibody LY3541860 that exhibits potent B cell inhibitory activities. LY3541860 inhibits B cell activation, proliferation, and differentiation of primary human B cells with high potency. LY3541860 also inhibits human B cell activities in vivo in humanized mice. Similarly, our potent anti-mCD19 antibody also demonstrates improved efficacy over CD20 B cell depletion therapy in multiple B cell–dependent autoimmune disease models. Our data indicate that anti-CD19 antibody is a highly potent B cell inhibitor that may have potential to demonstrate improved efficacy over currently available B cell–targeting therapies in treatment of autoimmune conditions without causing B cell depletion.

## Graphical abstract

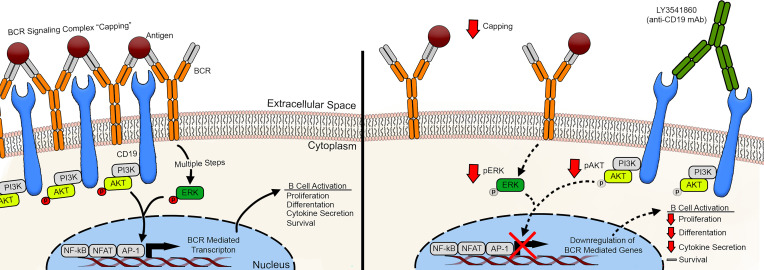

## Introduction

B cells play an important role in the development and progression of multiple autoimmune diseases. There has been tremendous clinical success of B cell–targeting therapies such as rituximab, ocrelizumab, belimumab, and inebilizumab, which have been approved for treatment of rheumatoid arthritis (RA), multiple sclerosis (MS), systemic lupus erythematosus (SLE), and neuromyelitis optica (NMO) (1). These B cell–targeting therapies work via either direct B cell depletion (rituximab, ocrelizumab, inebilizumab) or by neutralizing B cell survival factor (belimumab).

Despite the success of B cell–depleting therapies in autoimmune diseases, there are limitations that may explain why current B cell–depleting therapies demonstrate a range of outcomes depending on the disease and even among patients with the same disease. Multiple observations suggest that B cell depletion is not always sustained or complete. This is particularly true for the tissue-resident B cells, as demonstrated for the lymph node (2) and synovial B cells in RA patients (3, 4). This phenomenon has been further explored in the mouse model of SLE (5), where it has been demonstrated that depletion of B cells in tissues was impaired in autoimmune-prone mice compared with the healthy WT animals, even though peripheral blood B cells were effectively depleted in both cases. The data suggest that the mechanisms leading to the efficient cell depletion, such as ADCC and induction of apoptosis, are impaired in tissues in autoimmune conditions. These findings, together with clinical observations, suggest that B cell depletion in tissues might be incomplete in patients with autoimmune disorders. Therefore, novel nondepleting B cell–targeting therapies could demonstrate efficacy superior to the B cell–depleting therapies in autoimmune settings.

The majority of currently approved B cell–targeting therapies including rituximab and ocrelizumab deplete B cells via targeting CD20. Despite the success of anti-CD20–mediated B cell depletion, some data suggest targeting B cells via different surface receptors may provide additional benefits. For instance, CD19 has been highlighted as an attractive target due to its wider range of expression in B cells at different activation and developmental stages (6). Thus, during B cell development, expression of CD19 starts prior to the expression of CD20 at the late pro–B cell stage and is maintained throughout B cell activation and differentiation until differentiation into plasma cells (PCs), which is then associated with partial or complete loss of CD19 expression. In contrast, expression of CD20 does not start until later in the development at the pre–B cell stage and is lost partially or completely at the plasmabast differentiation stage (6). Therefore, targeting B cells via CD19 may allow for broader targeting of B cells, including CD19^+^CD20^lo^ plasmablasts, and this targeting is particularly attractive for multiple autoimmune indications. The presence of pathogenic plasmablasts with low CD20 and high CD19 expression has been reported in RA, SLE, MS, and other autoimmune conditions, and this B cell population has been demonstrated to be refractory to the anti-CD20–mediated B cell depletion (7–9)

In addition to being an attractive antigen for B cell targeting, CD19 has been previously described to play a role in the regulation of the B cell response. Thus, it has been demonstrated that cross-linking of CD19 leads to the inhibition of B cell proliferation (10, 11). CD19 has also been demonstrated to control TLR9 response in human B cells (12). At the molecular level, CD19 has been reported to play an essential role for B cell activation via promoting B cell receptor microcluster formation (13). CD19 deficiency in human and in mice has been associated with impaired humoral immune response, while CD19 overexpression resulted in autoimmune diseases (14–17). Taken together, these data suggest that CD19 is a critical regulator of B cell activation and function. Therefore, we hypothesized that cross-linking of CD19 may lead to the impairment of CD19 function, therefore leading to the inhibition of B cell activation and differentiation, and may provide a mechanism for inhibiting B cells in autoimmune conditions.

Here we describe an anti-CD19 B cell–inhibiting, nondepleting antibody LY3541860. Our data demonstrate that LY3541860 selectively binds to human CD19 on the surface of B cells and inhibits B cell function without causing B cell depletion. Treatment of B cells with LY3541860 leads to potent inhibition of primary B cell proliferation, activation, and differentiation. Furthermore, LY3541860 doesn’t have activity in ADCC/CDC or B cell apoptosis assays, which has been further confirmed in vivo using humanized NSG mice and cynomolgus monkeys. We have also identified a mechanism of action of anti-CD19 antibody that interferes with BCR capping (18) and signaling, suggesting an important role of CD19 in this process. Moreover, anti-mCD19 nondepleting antibody demonstrated improved efficacy over anti-CD20 B cell–depleting antibodies in different mouse B cell–dependent autoimmune disease models. Collectively, our data suggest that anti-CD19 mAb LY3541860 may represent a novel approach to targeting B cells in autoimmune conditions without inducing long-lasting B cell depletion.

## Results

### Generation and optimization of LY3541860.

**Figure 1:**
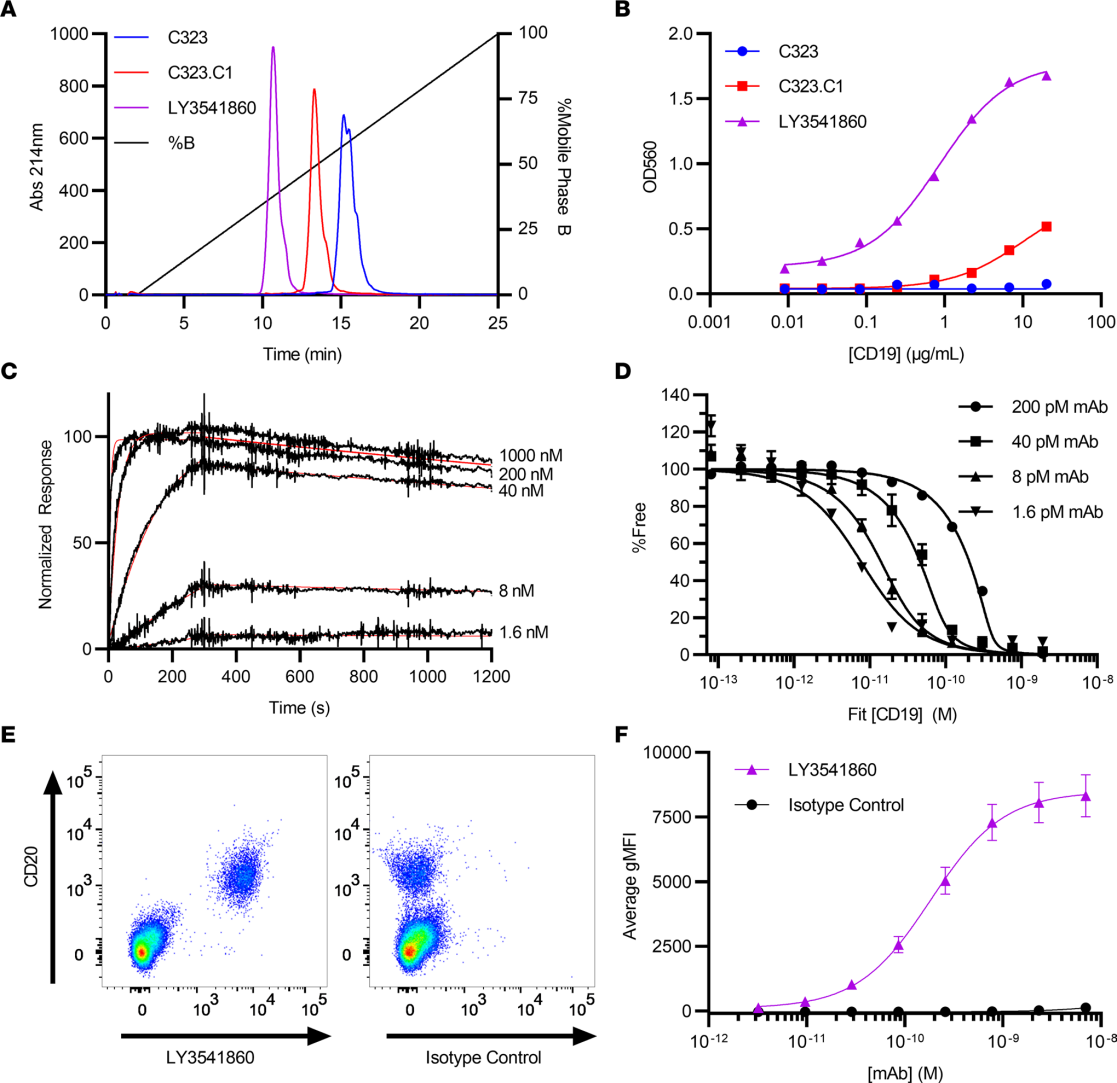
Generation, optimization, binding affinity, kinetics, and specificity of LY3541860. (**A**) Analytical HIC HPLC chromatograms of C323, C323.C1, and LY3541860 demonstrate the reduction in hybrophobicity through engineering. (**B**) ELISA binding to a titration of biotinylated huCD19-HSA fusion protein with C323 showing barely detectable binding under these conditions. (**C**) Representative SPR sensorgrams of LY3541860 Fab binding to human CD19-Fc are shown in black, and the 1:1 kinetic fit is shown in red. (**D**) Representative SET titrations of CD19 expressing cells at different fixed LY3541860 antibody concentrations. (**E**) Representative data demonstrating binding of fluorescently labeled isotype control antibody (right) or LY3541860 (left) in human whole blood (gated on viable CD45^+^ cells). (**F**) Dose response binding of fluorescently labeled LY3541860 and isotype control antibody on human B cells in whole blood (B cells gated as viable/CD45^+^/CD20^+^/CD3^–^) obtained from 4 independent donors. Data are shown as mean gMFI ± SEM.

We sought to discover a highly specific, high-affinity CD19 antibody with suitable physiochemical properties to be developed as a therapeutic. To identify antibodies with specificity for CD19, we employed a cell-based phage panning approach using HEK-293 coexpressing CD19 and CD21. The initial Fab hit from phage panning, C323, was confirmed by ELISA using the CD19 extracellular domain (ECD) protein, converted to IgG4, and then was confirmed to bind CD19 on cells by FACS using both Daudi human Burkitt’s lymphoma cell line and primary human B cells. C323 was subsequently optimized to reduce hydrophobicity (Figure 1A), improve affinity (Figure 1B), and revert select CDR positions to IGHV1-69 and IGKV3-20 germline to yield LY3541860, a high-affinity antibody with optimized biophysical properties and high germline sequence identity.

### Binding affinity and specificity of LY3541860.

The monovalent affinity and kinetics of the Fab fragment of LY3541860 binding to human (Figure 1C) and cynomolgus CD19 were measured by surface plasmon resonance (SPR) with average dissociation constants of 76.8 pM and 94.5 nM, respectively. The apparent equilibrium binding affinities (K_D_) of LY3541860 to membrane-bound human (Figure 1D) and cynomolgus monkey CD19 stably expressed on CHO cells was measured by Meso Scale Discovery solution equilibrium titration (MSD-SET), with average dissociation constants of 3.35 and 45.2 pM, respectively. These data are summarized in Table 1 and demonstrate both the high affinity of LY3541860 for CD19 as well as the potential for significantly increased apparent affinity to cell-surface CD19 due to avidity from bivalent engagement.

To determine the specificity and potency of binding of LY3541860 to human B cells in more physiologically relevant conditions, we evaluated binding in human whole blood using flow cytometry. The results indicated that LY3541860 binds exclusively to B cells in human whole blood (Figure 1E). No binding was observed on viable CD20^–^ non–B cell populations. The average EC_50_ from 2 independent experiments using a total of 4 different donors was 0.184 ± 0.008 nM (Figure 1F). Isotype control antibody showed no binding to B cells at any tested concentration.

Taken together, these data indicate that LY3541860 is a highly selective anti-CD19 antibody that both binds human CD19 with high affinity and selectively binds human B cells in the context of whole blood.

### LY3541860 does not induce ADCC, CDC, or B cell apoptosis.

Most B cell–targeting antibodies approved for the treatment of autoimmune conditions act by depleting B cells via induction of antibody-dependent cellular cytotoxicity (ADCC), complement-dependent cytotoxicity (CDC), B cell apoptosis, or a combination of these mechanisms (1). In contrast, LY3541860 is a B cell inhibitory antibody that inhibits B cell function without causing B cell depletion. Therefore, we evaluated the ability of LY3541860 to induce ADCC, CDC, or B cell apoptosis in vitro.

**Figure 2:**
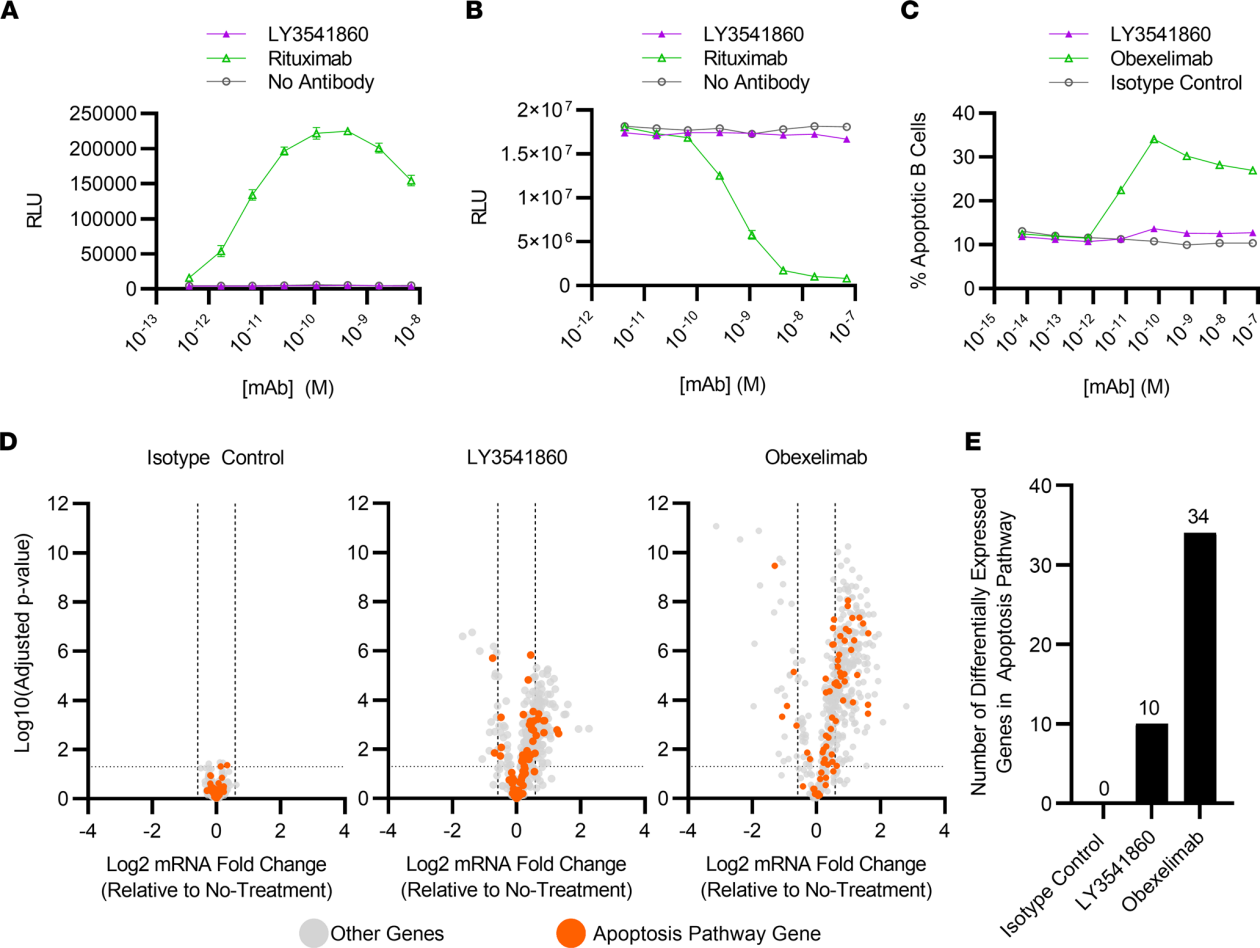
LY3541860 does not induce B cell apoptosis or cause ADCC/CDC in vitro. (**A**) In vitro ADCC assay. The data are representative of 3 assay runs. (**B**) In vitro CDC assay. The data are representative of 3 assay runs. (**C**) Induction of B cell apoptosis in vitro. Apoptotic B cells were detected using annexin V staining (gated as live, annexin V^+^). The assay was repeated 2 times using B cells from 4 independent donors. Data obtained from 1 representative donor are shown. Each point represent mean ± SEM from 3 technical replicates. (**D**) Volcano plots showing differential expression of apoptosis pathway genes by B cells treated in vitro with LY3541860, isotype control, or obexelimab. mRNA was counted on the NanoString nCounter platform (*n* = 6 per groups). (**E**) Bar plots quantifying the number of differentially expressed apoptosis pathway genes by B cells treated in vitro with LY3541860, isotype control, or obexelimab.

In vitro ADCC and CDC assays were performed using rituximab, a potent inducer of ADCC and CDC response (19), as a positive control. The results shown in Figure 2, A and B, indicate that LY3541860 demonstrated no activity in either CDC or ADCC assays at the tested concentrations, suggesting that it is unlikely to exhibit CDC or ADCC activity in vivo.

Some B cell–targeting antibodies are known to induce B cell apoptosis in addition to the ADCC and CDC activity (19, 20), which can contribute to B cell depletion in vivo. Therefore, we evaluated the ability of LY3541860 to induce apoptosis in primary human B cells. As demonstrated in Figure 2C and Supplemental Figure 1 (supplemental material available online with this article; https://doi.org/10.1172/jci.insight.166137DS1), LY3541860 demonstrated minimum to no apoptosis induction in primary human B cells compared with the isotype control antibody at all tested concentrations. Obexelimab (XmAb5871), a B cell–targeting antibody, is known to reduce B cell numbers in clinical studies (21, 22) and was demonstrated to induce B cell apoptosis in vitro (20), was used as a positive control, and demonstrated significantly increased frequency of apoptotic B cells in a dose-dependent manner in the same assay. We further explored the difference between LY3541860 and obexelimab, measuring their ability to induce gene expression changes associated with B cell apoptosis. As demonstrated in Figure 2, D and E, LY3541860 induced significantly fewer genes associated with apoptosis pathways compared with obexelimab, confirming our finding. These results indicate that LY3541860 does not induce primary human B cell apoptosis in vitro, unlike another anti-CD19 antibody obexelimab.

In concert, the data suggest that LY3541860 is unlikely to induce B cell depletion or reduce B cell counts in vivo.

### LY3541860 inhibits B cell proliferation in vitro.

So far, our data indicate that LY3541860 is a high-affinity anti-CD19 antibody that selectively binds human B cells and is unlikely to induce B cell depletion or reduction in B cell counts in vivo.

**Figure 3:**
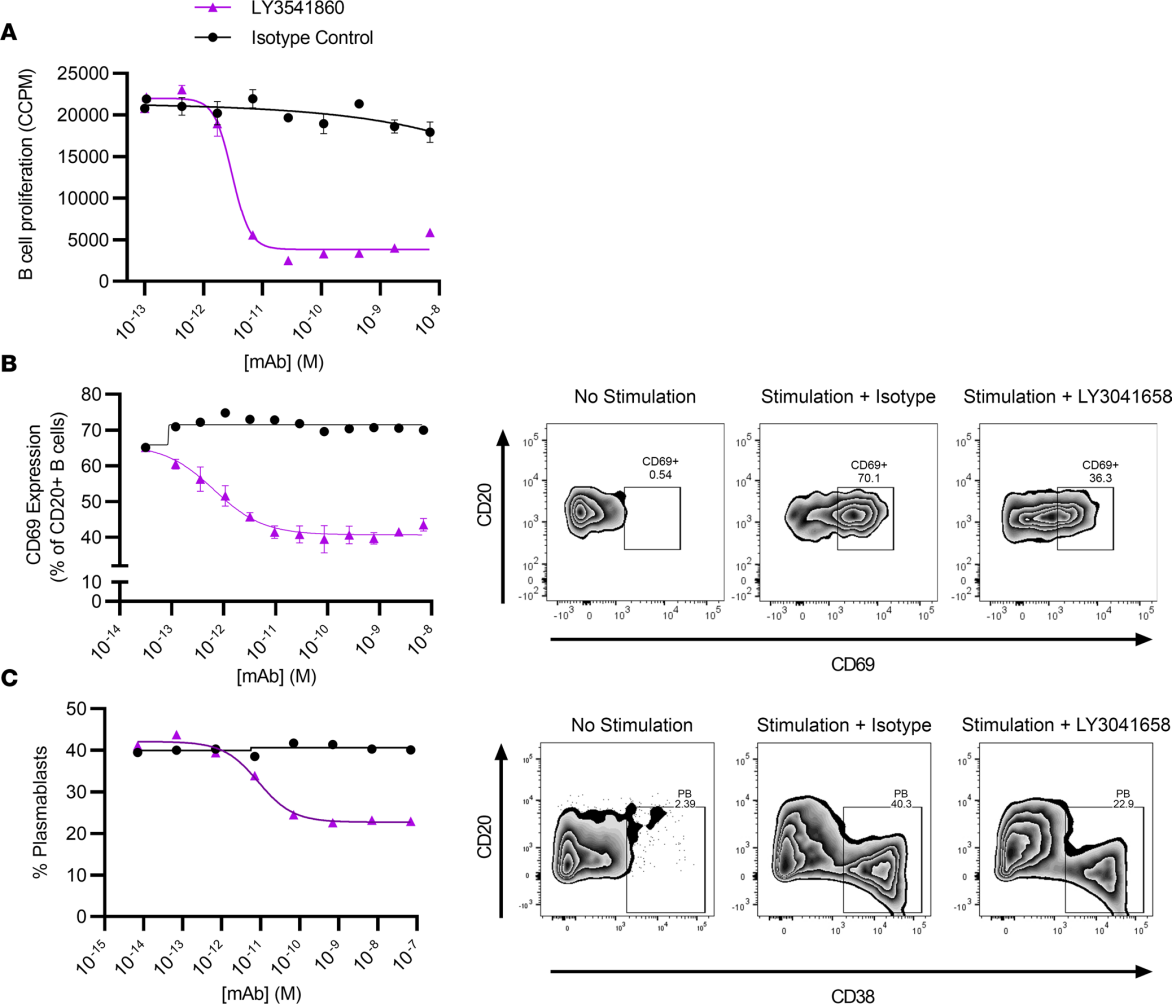
LY3541860 inhibits B cell proliferation, activation, and plasmablast differentiation in vitro. (**A**) Representative data showing a concentration-dependent decrease of B cell proliferation induced by anti-IgM cross-linking in vitro IC_50_, determined by generating dose-response curves and fitting to a 4 parameter logistic fit of the CCPM values as a function of LY3541860 concentration (mean ± SEM, *n* = 3). (**B**) Inhibition of CpG-induced upregulation of CD69 on B cells in human whole blood. Representative data showing a concentration-dependent decrease of percent of CD69^+^ cells on viable B cells in human whole blood after incubation with CpG for 24 hours. The IC_50_ was determined by generating dose response curve and fitting to a 4 parameter logistic fit of the percent of CD69^+^ cells as a function of LY3541860 concentration. Data are shown as mean ± SEM. (**C**) Inhibition of primary memory B cell differentiation into plasmablasts in the presence of BAFF/IL-21/IL-2/anti-CD40 and different concentrations of either LY3541860 or isotype control as indicated. The graph shows the percent of plasmablasts (identified as CD20^lo^/CD38^hi^) in culture. The assay was performed 3 times and data from one representative experiment is shown.

Next, we evaluated the inhibitory activity and potency of LY3541860 in vitro using primary human B cells. Cross-linking of CD19 has been previously demonstrated to inhibit B cell activation (10, 11); therefore, we asked whether LY3541860 is capable of reducing BCR-mediated B cell proliferation in vitro. To address this question, primary human B cells were isolated from healthy volunteers, and proliferation was triggered by induction of surface IgM clustering on human B cells. Figure 3A and Supplemental Figure 2 demonstrate a LY3541860 concentration–dependent reduction of B cell proliferation. The isotype control did not inhibit B cell proliferation at any tested concentrations (Figure 3A). LY3541860 was tested in the B cell proliferation assay using B cells obtained from 16 independent donors (Supplemental Table 1). The average IC_50_ was calculated to be 0.008 ± 0.001 nM (mean ± SEM, *n* = 16), indicating that treating B cells with LY3541860 can impair BCR-mediated B cell proliferation.

We have confirmed the results using different methods of detection for B cells proliferation; as demonstrated in Supplemental Figure 2, similar results were obtained. In addition, we confirmed that inhibition of B cell proliferation was not associated with increased cells death (Supplemental Figure 2), further supporting the nondepleting, B cell–inhibiting nature of LY3541860.

### LY3541860 inhibits CpG-induced B cell activation in human whole blood.

Our data indicate that LY3541860 can inhibit B cell activation induced by the cross-linking of surface BCR. However, in addition to BCR cross-linking, B cells are also known to be activated by the innate stimuli including TLR agonists (23).

It has been previously described that CD19 is required for TLR9-induced B cell activation (12) and cross-linking of CD19 can inhibit CpG-induced B cell activation (24). Therefore, next we asked whether LY3541860 could also affect activation of B cells caused by stimulation with CpG in the context of human whole blood; B cell activation in these conditions can be detected by upregulated expression of B cell activation markers such as CD69, CD80, and CD86. We did not observe significant induction of CD80 in these experiments; induction of CD86 was observed in some but not all donors. Therefore, we focused on CD69 expression, which was consistently upregulated in all tested donors. As demonstrated in Figure 3B, LY3541860 dose-dependently inhibited expression of CD69 on B cells upon CpG-induced activation, while isotype control did not demonstrate any effect on CD69 expression level at any tested concentrations (Supplemental Figure 3). The inhibitory effect of LY3541860 was tested using whole blood from 4 independent healthy donors. Supplemental Table 2 shows the average IC_50_ of LY3541860 from 2 independent experiments using a total of 4 different donors was 0.006 ± 0.003 nM (mean ± SEM, *n* = 4). Thus, LY3541860 inhibited both BCR- and CpG-induced activation of primary human B cells, indicating that LY3541860 can inhibit both BCR- and TLR-induced human B cell activation.

### LY3541860 inhibits memory B cell differentiation into plasmablasts.

Plasmablasts are antibody-secreting cells that play critical roles in autoimmunity due to their ability to produce autoantibodies, and CD19 is highly expressed on plasmablast cells (25). Differentiation of B cells into plasmablasts is critical for the pathogenesis of autoimmune diseases, and a successful autoimmune therapy should be able to inhibit this process.

Therefore, the ability of LY3541860 to inhibit memory B cell differentiation into plasmablasts was next tested in vitro. In contrast to previously published data using a different anti-CD19 antibody (24), LY3541860 dose-dependently inhibited differentiation of primary human memory B cells into plasmablasts (Figure 3C and Supplemental Figure 4). Isotype control did not inhibit plasmablast differentiation at any concentration tested. This result indicates that, in addition to inhibiting B cell activation and proliferation, LY3541860 also inhibits differentiation of primary human memory B cells into plasmablasts, and this should ultimately affect immunoglobulin production.

### LY3541860 inhibits B cell function in vivo.

**Figure 4:**
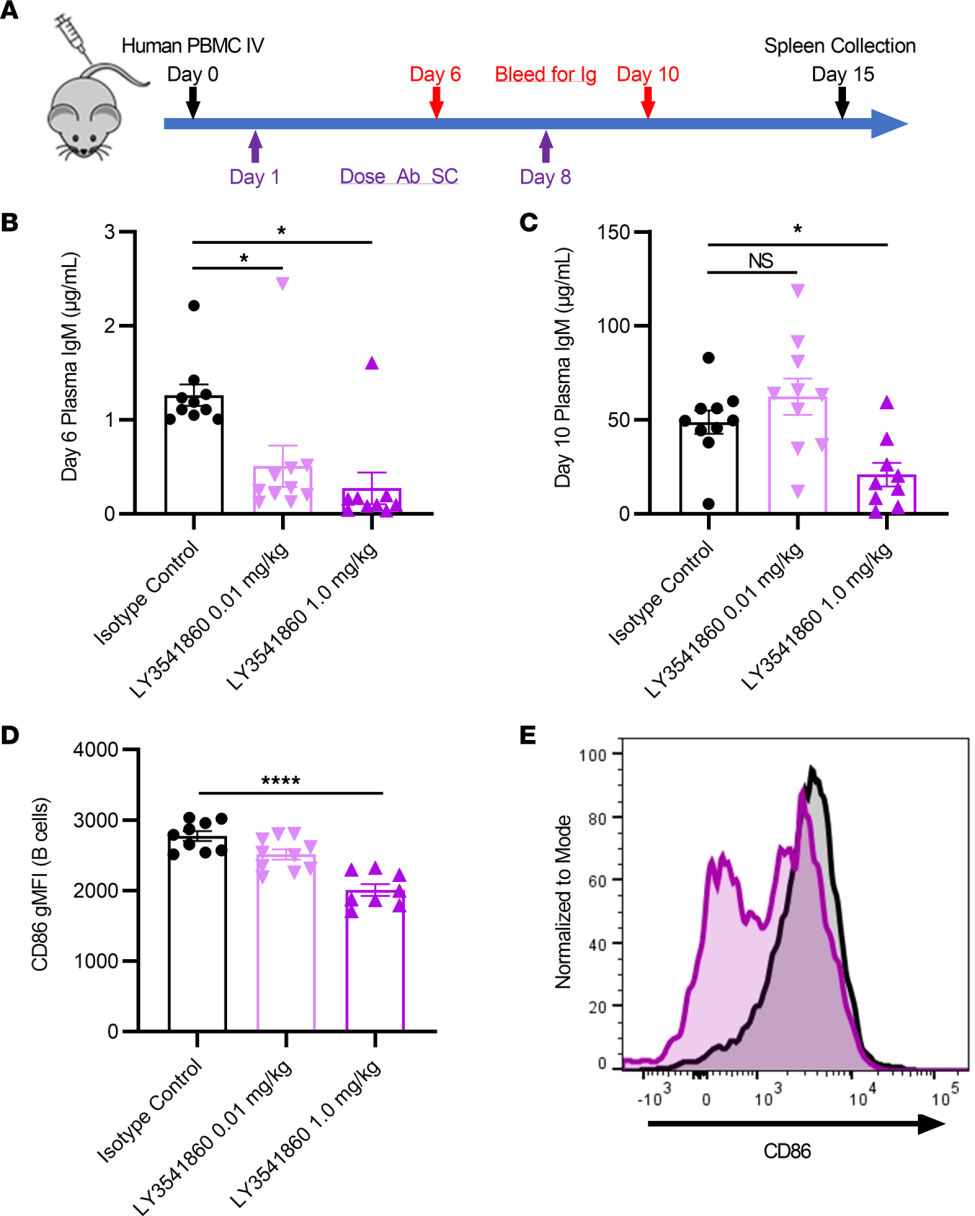
Inhibition of B cell activation in vivo in humanized NSG mice. (**A**) Study design. (**B** and **C**) Concentration of human IgM in plasma of hNSG mice treated with either isotype or different doses of LY3541860 as indicated on day 6 (**B**) or 10 (**C**) after engraftment. (**D**) Expression of CD86 on human B cells in spleens of hNSG mice on day 10 after engraftment. *P < 0.05 versus isotype by 1-way ANOVA with Tukey’s post hoc test; data are shown as mean ± SEM. *n* = 9, 8, and 10 for the isotype, 1.0, and 0.1 mg/kg LY3541860 groups, respectively. (**E**) Representative FACS histogram demonstrating expression of CD86 on human B cells in spleens of hNSG mice on day 10 after engraftment. Isotype treated, black; 1.0 mg/kg LY 3541860 treated, magenta.

In order to evaluate the ability of LY3541860 to inhibit immunoglobulin production and B cell activation in vivo, a humanized mouse model in which human PBMCs were engrafted into immunocompromised mice was used (Figure 4A).

Injection of human PBMCs into NOD scid γ (NSG) mice resulted in marked engraftment of functional B cells as measured by secretion of human IgM into the periphery and immunophenotyping of splenocytes, as previously described (26). Human IgM in animals treated with isotype control antibodies increased rapidly from nondetectable to 1.3 ± 0.1 μg/mL and 48.8 ± 6.2 μg/mL on days 6 and 10 after engraftment, respectively. LY3541860 administered at 0.01 and 1.0 mg/kg/week significantly attenuated IgM secretion by 60% and 78%, respectively, on day 6 as shown in Figure 4B. A 57% reduction in circulating IgM was also observed on day 10, with the 1.0 mg/kg/week dose of LY3541860 (Figure 4C). Activation of human B cells in splenocytes obtained from the engrafted NSG mice was determined by the expression of CD86, a common B cell activation marker; other activation markers, such as CD80 and CD69, were explored but demonstrated relatively low expression in this model at this time point. As illustrated in Figure 4, D and E, treatment with LY3541860 dose-dependently reduced the expression of CD86 on human B cells. These results demonstrate that LY3541860 dose-dependently inhibited IgM secretion and B cell activation in vivo in a humanized mouse model of B cell function.

### LY3541860 interferes with surface BCR capping and inhibits BCR-induced signaling.

Functional characterization of LY3541860 indicates that this anti-CD19 antibody inhibits multiple aspects of B cell activation and differentiation. Such functional activity is not widely expected for an anti-CD19 antibody, and only a few published reports indicate similar activity of other anti-CD19 antibodies (10, 11, 24), despite CD19 having been studied for several decades. Therefore, the molecular mechanism of B cell inhibition caused by the LY3541860 binding to the CD19 required further investigation.

CD19 is a well-known B cell marker. It serves as a part of the B cell receptor complex and provides amplification of B cell receptor signaling by bringing signaling components in proximity with the rest of the BCR subunits (27).

As previously demonstrated by Depoil et al. (13), CD19 is essential for the formation of microclusters and assembly of the BCR “signalosome” or so called “capping” of BCR (18). We hypothesized that binding of LY3541860 to CD19 on the B cell surface interferes with the ability of CD19 to promote BCR capping, leading to the distribution of BCR on the surface of the B cell membrane. Fluorescence microscopy was used to investigate this hypothesis.

**Figure 5:**
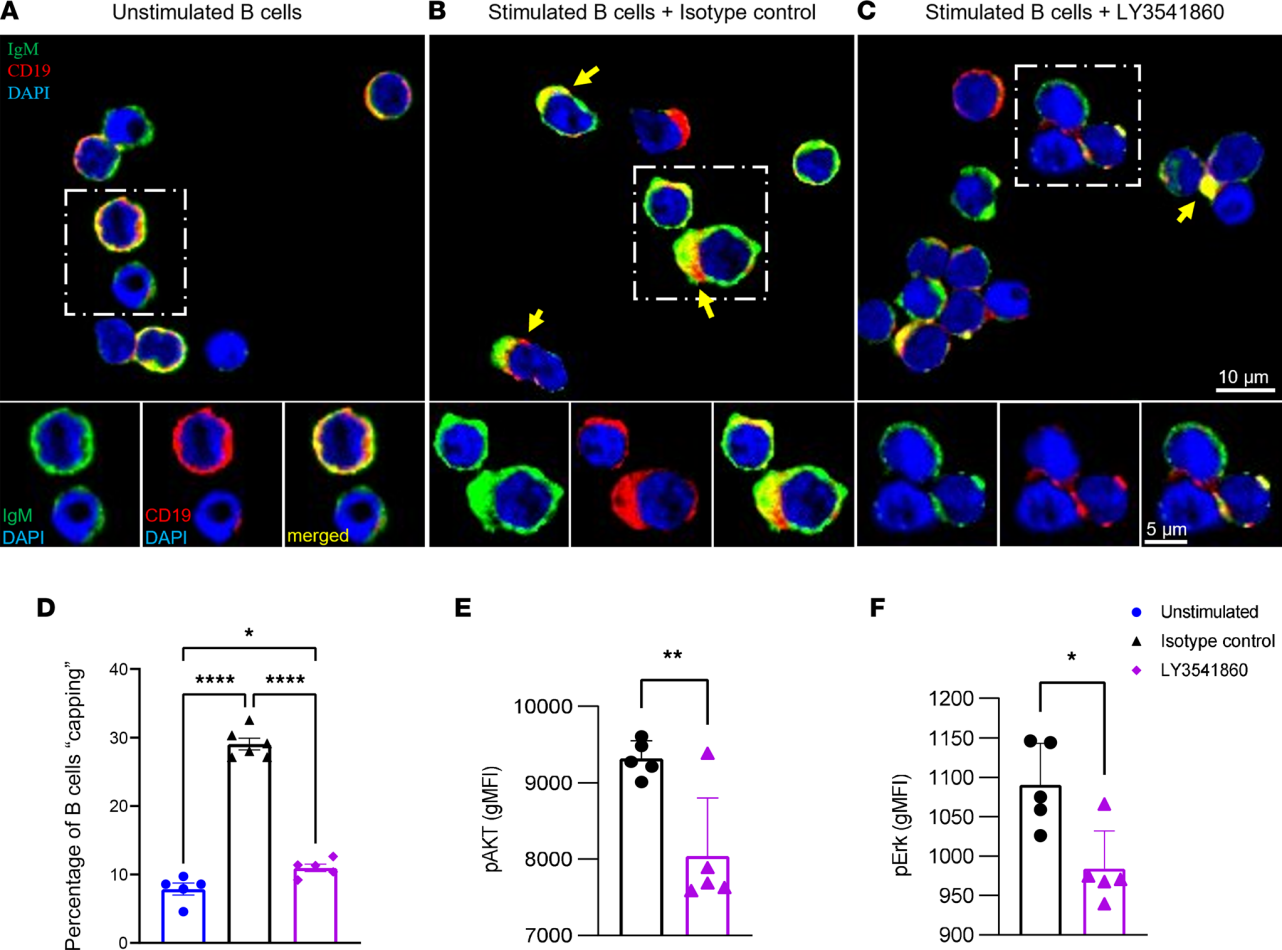
Inhibition of BCR capping and downstream BCR signaling with LY3541860 treatment. (**A**) Immunofluorescence images showing primary human B cells in the absence of stimulation. CD19 is shown in red, IgM in green, and nuclear staining in blue. (**B**) Immunofluorescence images showing primary human B cells after the overnight isotype control treatment followed by 24-hour stimulation. CD19 is shown in red, IgM in green, and nuclear staining in blue. Yellow arrows indicate capping. Dotted line box is shown in higher magnification. . (**C**) Immunofluorescence images showing primary human B cells after the overnight LY3541860 treatment followed by 24-hour stimulation. CD19 is shown in red, IgM in green, and nuclear staining in blue. Yellow arrows indicate capping. Dotted line box is shown in higher magnification. Scale bars: 5 μm (insets), 10 μm (merged image). (**D**) Percentage of untreated unstimulated or stimulated B cells “capping” after the LY3541860 or Isotype control treatment. *n* = 6 for isotype control (total of 585 cells) and *n* = 5 for LY354186 (total of 1933 cells) of randomly imaged coverslip regions. *P = 0.0475, ****P < 0.0001; 1-way ANOVA with Tukey’s test. (**E** and **F**) gMFI of pAKT (**E**) and pERK (**F**) on B cells activated in the presence of LY3541860 or isotype control; data are shown as mean ± SEM. Experiment was performed 3 times using B cells from 5 healthy donors. Representative data from 1 donor are shown.

IgM and CD19 are evenly distributed on the surface of primary human B cells in the absence of B cell stimulation, as shown in Figure 5A. As expected, stimulation via BCR induced appreciable capping of the surface BCR and CD19 (Figure 5B). Interestingly, B cells stimulated via BCR in the presence of LY3541860 demonstrated significantly fewer capping events, showing that binding of LY3541860 inhibits the ability of CD19 to promote the assembly of these structures on the B cell surface (Figure 5, C and D). LY3541860 treatment did not show an effect on resting (unstimulated) B cells (Supplemental Figure 5). We observed remarkably similar donor-to-donor efficacy of LY3541860, across 5 donors, and it was also independent of treatment duration (Figure 5, B and C, and Supplemental Figure 6, A–C). These data are in agreement with a previously published observation suggesting that CD19 deficiency causes defects in B cell activation due to inefficient formation of microclusters (13). Taken together, these data strongly suggest that LY3541860 inhibits B cell activation by interfering with the efficient BCR clustering and capping of BCR on B cell surface.

Next, we hypothesized that LY3541860-induced inefficient capping of BCR may result in inefficient downstream BCR signaling. Phosphorylation of central components of the BCR signaling pathway were explored by flow cytometry. B cell activation in the presence of LY3541860 binding resulted in the reduced phosphorylation of ERK1/2 and AKT1 (Figure 5, E and F). These results support previously published data demonstrating that CD19 is necessary for efficient activation of Akt following B cell activation via surface BCR (28) and enhances activation of ERK2 (29).

Collectively, these data demonstrate that LY3541860 interferes with the efficient capping of BCR and downstream signaling, which results in overall inhibition of B cell function.

### LY3541860 does not induce B cell depletion in cynomolgus monkeys.

To further confirm the nondepleting nature of LY3541860, its effect on B cell levels in cynomolgus monkeys was evaluated. Cynomolgus monkeys were given a single i.v. (1 mg/kg) or s.c. (10 mg/kg) injection of LY3541860. Target engagement was measured by analyzing changes in the amount of unoccupied CD19 over time in CD45^+^CD20^+^ B cells in blood. The results indicate > 97% and > 80% target occupancy at 168 hours and 504 hours after administration, respectively, indicating nearly complete and sustained target engagement by LY3541860 at both doses (Table 2). Moreover, the data indicate that there was no significant time- or dose-dependent change in B cell frequency (Table 2). Taken together, the data show that LY3541860 does not induce B cell depletion in vivo with sustained CD19 target engagement.

### Anti-mCD19 nondepleting antibody demonstrates improved efficacy in mouse autoimmune models compared with anti-mCD20 B cell–depleting antibodies.

Next, we asked whether B cell inhibition via targeting CD19 with antibody can provide benefits for treatment of autoimmune diseases. In addition, we investigated whether such an approach could provide additional efficacy when compared with already-approved B cell–targeting therapeutic mechanisms such as anti-CD20–mediated B cell depletion. To investigate the effect of anti-CD19 mAb in multiple B cell–dependent autoimmune disease models, an anti-mCD19 antibody with binding affinity and functional activity closely representative to LY3541860 was generated (Supplemental Figure 7).

B cell–depletion therapy was proven to be efficacious in RA and MS, and rituximab has demonstrated promising results in type 1 diabetes (T1D) (30); therefore, the efficacy of anti-mCD19 nondepleting and anti-mCD20 depleting antibodies was compared in various mouse autoimmune disease models: arthritis, MS, and TD1. The anti–mCD20 18B12 mAb, commonly used for in vivo B cell depletion, was used in these studies (5).

**Figure 6:**
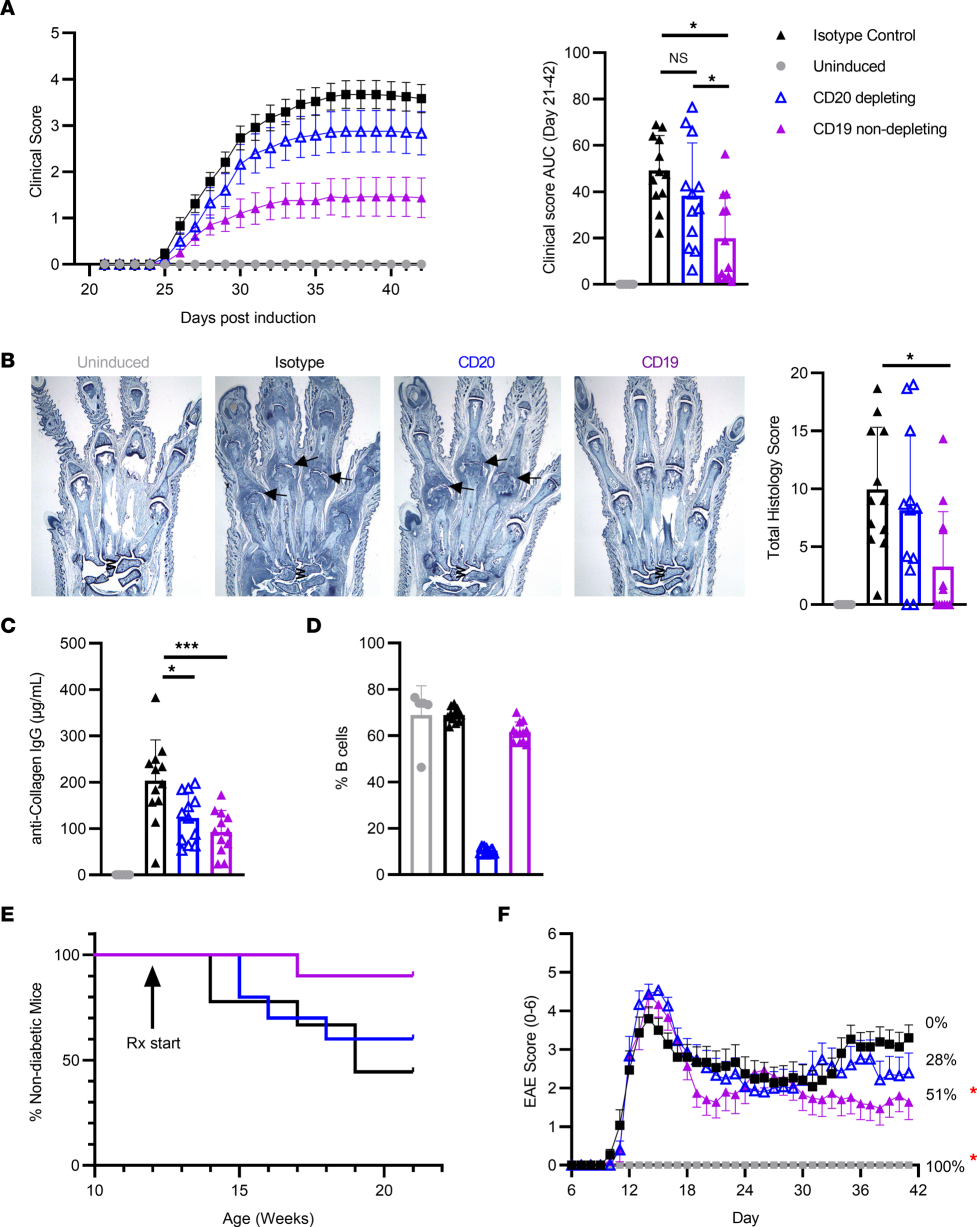
Improved efficacy of surrogate anti-CD19 antibody over CD20-mediated B cell depletion in mouse models of autoimmunity. (**A**–**C**) CIA model, *n* = 12 mice/group representative of 3 independent experiments. (**A** and **B**) Clinical score over time and histology microphotographs of the front paws and summary histology score. Arrows indicate affected joints with severe inflammation and cartilage damage, with marked pannus and bone resorption as well as moderate periosteal bone formation. (**C**) Anti–collagen IgG levels in CIA model (day 42). (**D**) Frequency of B cells (live/CD45^+^/B220^+^/CD3^–^) in spleens, day 42. (**E**) NOD model (*n* = 10 mice/group), incidence of the disease over time (representative of 2 independent experiments). (**F**) EAE model (*n* = 15 mice/group), clinical score over time. Percent of inhibition over isotype control on the last day of study is indicated. Red asterisk indicate statistical significance over isotype control.

First, we explored the efficacy of the anti-mCD19 antibody in the collagen-induced arthritis (CIA) model. In this model, mice immunized with type II collagen develop autoimmune arthritis that shares clinical and histological features with RA (31). In our study, isotype control–treated mice immunized with collagen developed observable signs of joint inflammation starting at day 25, while isotype-treated nondiseased (nonimmunized) mice had no observable joint inflammation throughout the study (Figure 6A). Treatment starting on day 19 with an anti-mCD20–depleting antibody ameliorated average clinical scores, whereas treatment with the anti-mCD19 resulted in further reduction of average clinical scores (Figure 6A). Anti-mCD20 B cell depletion did not significantly reduce clinical score AUC or histology scores for all joints compared with isotype control (Figures 6, A and B). On the other hand, the anti-mCD19 significantly reduced clinical score AUC compared with isotype control and also significantly reduced histology scores for all joints (Figure 6, A and B). Associated with the reduction in disease severity with the anti-mCD19 treatment, plasma anti–collagen IgG levels were also significantly reduced compared with the isotype control to a greater extent than anti-CD20 treatment, further suggesting inhibition of B cell function in this model (Figure 6C). These results demonstrate that nondepleting anti-mCD19 antibody reduced disease severity to a greater extent than the anti-mCD20 depleting antibody, as assessed by clinical score AUC in the CIA model of mouse arthritis. We have also confirmed that mouse B cells were intact in mice treated with anti-mCD19, while significant depletion was observed in anti-mCD20–treated animals (Figure 6D).

Next we tested anti-mCD19– and anti-mCD20–depleting antibodies in NOD mice, a well-established model of TD1. It has been demonstrated that B cells contribute to the development of the pathogenesis of the disease in NOD mice (reviewed in ref. 32). In this model, the anti-CD20 treatment demonstrated minimal to no efficacy in the reduction of disease incidence (Figure 6E). This result contradicts the previously published data suggesting that anti-mCD20 B cell depletion prevents autoimmune diabetes in NOD mice (33, 34). We would like to highlight that we have initiated our treatments when mice achieved 12 weeks of age (semitherapeutic mode), in contrast to the other published studies that initiated treatment significantly earlier (at 4 or 9 weeks of age). Since it has been demonstrated that B cell depletion at earlier stages of the disease provides greater efficacy than treatment at the later stages (33), we suggest that the lack of efficacy of anti-mCD20 B cell depletion in our study is attributed to a later initiation of the treatment than what was previously reported. The surrogate anti-mCD19 treatment, however, significantly reduced the incidence of disease over time, even when the treatment was initiated in 12-week-old animals (Figure 6E).

Next, we compared both antibodies in a proteolipid protein–induced (PLP-induced) relapsing-remitting experimental autoimmune encephalomyelitis (EAE) model of MS. The efficacy of CD20-mediated B cell depletion in the EAE model has been controversial, demonstrating protection, no efficacy, or even exacerbation of the disease depending on the study design (35, 36). In our study, anti-mCD20 treatment did not result in a beneficial effect in the EAE model at any time point; however, mice treated with the anti-mCD19 had a significantly reduced EAE score on days 35–41 compared with isotype control, with a 51% decrease in EAE score on the final day (Figure 6F). Flow cytometry analysis at the end of the study demonstrated no reduction in B cell frequency in anti-CD19–treated mice (data not shown).

It has been previously demonstrated that B cell depletion in tissues might be incomplete in the context of human and mouse autoimmunity. Therefore, it was important to evaluate the engagement of CD19 on tissue B cells upon treatment with this antibody. We were able to confirm that B cells obtained from different lymphoid organs had their CD19 completely occupied by the treatment antibody (data not shown).

Taken together, our data indicate that this high-affinity anti-mCD19 nondepleting antibody can improve efficacy in autoimmune diseases when compared with anti-CD20 B cell–depletion therapies, which are currently approved for treatment of multiple autoimmune diseases.

### Anti-CD19–induced inhibition of B cells is reversable upon washout from the B cell surface.

**Figure 7:**
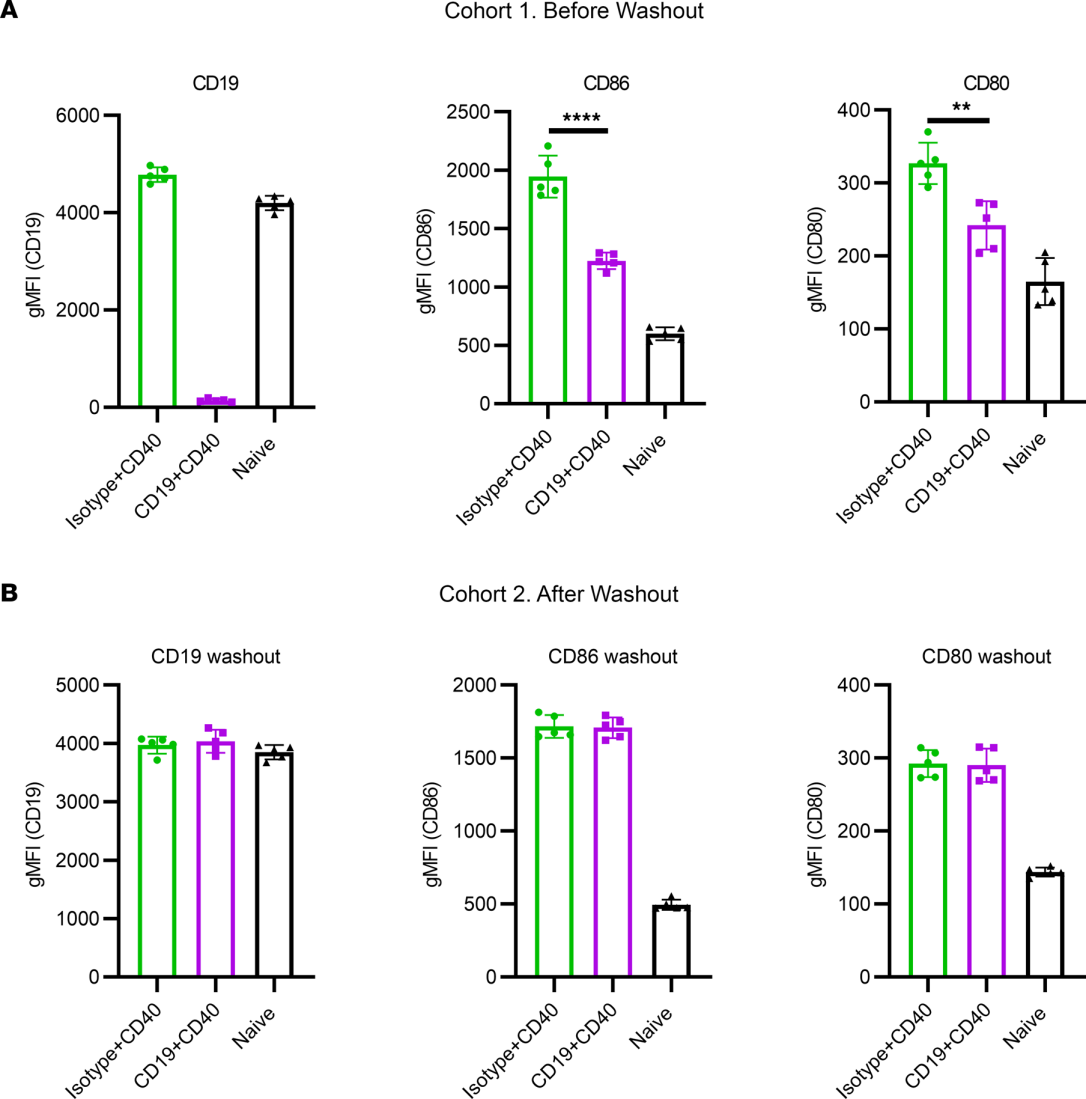
Reversibility of anti-CD19–induced B cell inhibition. Expression of “free CD19” (unoccupied by the treatment), CD86, and CD80 on splenic B cells after anti-CD40–induced B cell activation in vivo. (**A**) Anti-CD19 antibody introduced 24 hours before anti-CD40 stimulation (before washout). (**B**) Anti-CD19 antibody introduced 1 month before anti-CD40 stimulation (after washout). Data are shown as mean ± SEM, *n* = 5, representative of 2 independent experiments.

One of the major downsides of B cell depletion is a very slow restoration of the B cell compartment; this leads to long-lasting immune suppression and increased risk of infections. Therefore, we asked whether the inhibitory effect of nondepleting anti-mCD19 mAb is reversible. In order to investigate this question, we utilized an in vivo anti-CD40–induced B cell activation model. In this model, mouse B cells were activated in vivo by anti-CD40 agonistic antibodies and B cell activation could be measured by the elevated expression of activation markers such as CD80 and CD86. As demonstrated in Figure 7A, anti-CD19 mAb significantly reduced B cell activation in this model, and this activation was detected by downregulation of the expression of CD80 and CD86 on the B cell surface. Next we asked whether B cell inhibition can be reversed upon wash-out of anti-mCD19 mAb from B cell surface. For that purpose, mice were injected with anti-CD19 mAb and rested for 4 weeks, at which point the complete washout of anti-mCD19 mAb from the B cell surface was confirmed by FACS. Then mice were injected with anti-CD40 to induce B cell activation. In this experiment, there was no effect of prior anti-mCD19 treatment on the expression of activation markers (Figure 7B).

These data demonstrate that anti-CD19–induced B cell inhibition is reversible and B cells regain their function after the drug is washed out from the B cell surface. This is different from the kinetics of B cell depletion, which lasts for many months — often over a year.

## Discussion

Recent success of B cell–depleting therapies in clinical studies have revealed a previously underappreciated effect of B cells in autoimmune diseases (1). Such clinical success has expanded the understanding of the critical role of B cells, even in diseases like MS, which were historically viewed as T cell mediated. However, it is recognized that B cell–depleting therapies present certain limitations and fail in some patients and certain indications, including in patients with SLE. In addition, it has been demonstrated that patients receiving B cell–depleting therapies mount poor response to COVID-19 vaccines and experience greater complications when infected with COVID-19 (37). Therefore, it is possible that more efficacious B cell therapies not associated with long-lasting B cell depletion could be leveraged for the treatment of autoimmune diseases.

The goal for this study was to design a novel B cell–targeting therapy that would not cause reduction of B cell counts by either depleting B cells or inducing B cell apoptosis and that has a potential for improved efficacy. To achieve this goal, we have generated a nondepleting, highly potent anti-CD19 mAb, LY3541860, with picomolar affinity to human CD19 and suitable biophysical properties for development as a therapeutic. Functionally, LY3541860 inhibits B cell activation, proliferation, and differentiation with single-digit picomolar potency in primary human B cells. It is important to note that LY3541860 is able to inhibit B cell activation caused by a wide range of signals, including BCR cross-linking, innate stimulation via TLR9 (by CpG) in the context of human whole blood as well as cytokine and CD40-induced differentiation, which has been previously demonstrated not to be affected by another anti-CD19 antibody (24). These findings indicate that LY3541860 will be able to inhibit B cell activation caused by a variety of signals in the context of human autoimmunity, which is important since it has been demonstrated that BCR-, innate-, and cytokine/CD40-induced signaling all play roles in the development of autoimmunity.

It was also remarkable how LY3541860 can inhibit different outcomes of B cell activation, including B cell proliferation, induction of activation markers, and differentiation into plasmablasts. These processes play an important role in the progression of autoimmune disease, and LY3541860’s ability to inhibit these functional activities demonstrates promising therapeutic potential.

Moreover, data generated using a surrogate anti-mCD19 antibody confirms that treatment with such an antibody not only demonstrates efficacy in multiple autoimmune models without causing B cell depletion, but it also demonstrates improved efficacy over CD20-mediated B cell deletion. It is important to highlight that, in these studies, we only utilized 1 anti-CD20 clone, 18B12; therefore, it is possible that the efficacy of other anti-CD20–depleting clones might be different from the one we have explored in this manuscript. Nevertheless, our data suggest that this B cell–targeting therapy may provide more efficacious and safer options for the treatment of autoimmunity.

CD19 is a well-known B cell surface antigen that was first cloned in 1989 by T. Tedder (38) and, therefore, has been studied for over 3 decades. CD19 deficiency in humans leads to hypogammaglobulinemia and defective B cell responsiveness to antigenic stimulation (14, 16) as well as hyperresponsiveness to TLR stimulation (13, 27–29). Furthermore, CD19-KO mice were generated and described in 1995, providing additional information on the importance this receptor for B cell activation and function (14, 16). Thus, it became clear that CD19 is required for mounting an antibody response to T cell–dependent antigens. CD19-deficient mice demonstrated a lack of germinal center formation and affinity maturation of serum antibodies, and B cell proliferation and serum immunoglobulin levels were also impaired in the absence of CD19 expression. These observations revealed the importance of CD19 for B cell response in mice and humans; however, the molecular mechanism of how CD19 contributes to the B cell activation remained unclear.

In late 1990s early 2000s, several publications described an important role of CD19 for BCR-mediated signaling, providing additional insight on molecular mechanism of CD19 function (13, 27–29). Nevertheless, researchers of only a handful of publications have ever suggested the possibility of B cell inhibition by cross-linking CD19 (10, 27, 39). Therefore, we questioned why cross-linking of CD19 has not been used as a therapy for autoimmune disorders. We suggest that the answer to this question lies in the unique properties of LY3541860, an antibody that possesses excellent biophysical properties and very high affinity to CD19. We suggest that this tight binding of LY3541860 to CD19 leads to strong cross-linking of the antigen, which is necessary for efficient inhibitory activity.

**Figure 8:**
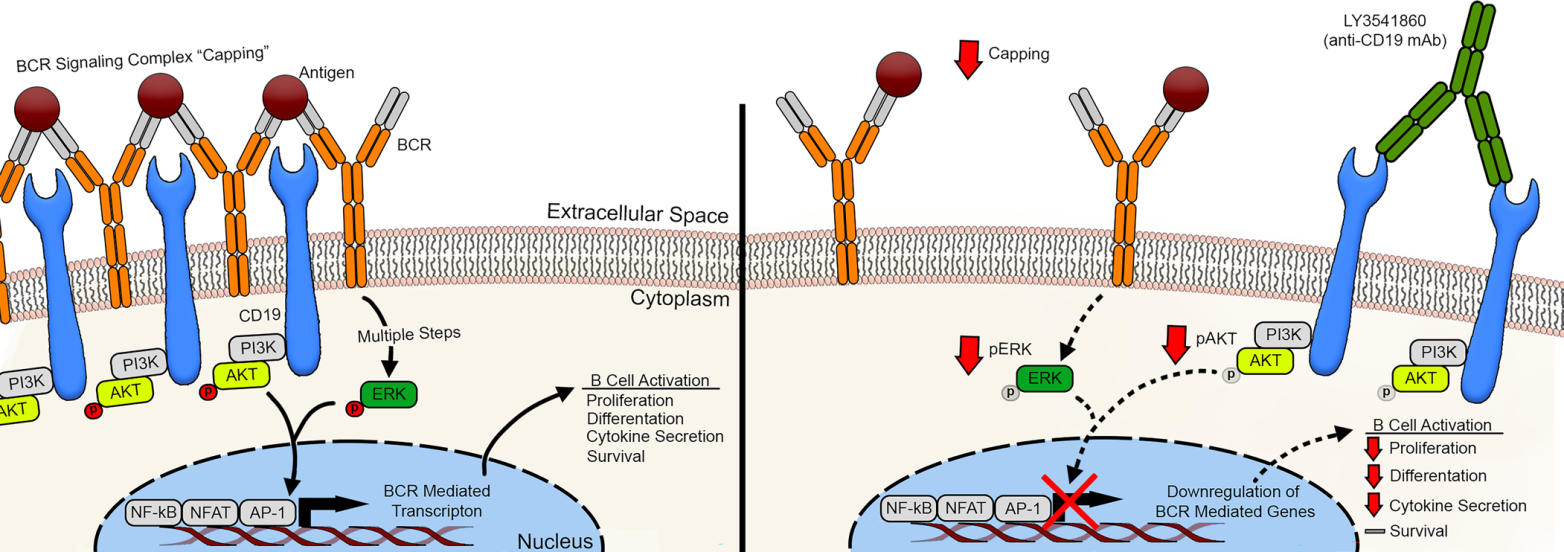
Model of the molecular mechanism of action of LY3541860. Left: CD19 facilitated BCR capping upon antigen recognition, leading to the formation of signalosome, which results in enhanced phosphorylation of downstream signaling components (Erk and AKT) and in enhanced transcription of BCR mediated genes and the induction of efficient B cell activation. Right: LY3541860 prevents CD19 from facilitating BCR capping, leading to the blunted phosphorylation of downstream signaling components, changes in the transcription of BCR-mediated genes, and inhibition of B cells activation.

Intrigued by the strong inhibitory activity of the LY3541860, we explored the molecular mechanism of action of this molecule. The results of our studies indicate that cross-linking of CD19 leads to the inefficient capping of BCR on the B cell surface, which in turn results in the blunt downstream signaling transduction (Figure 8). This mechanism of action closely resembles the phenotype observed with CD19 deficiency. In particular, it has been demonstrated that lack of CD19 leads to inefficient clustering of BCR (13), inhibition of phosphorylation of downstream signaling molecules (27), and blunted B cell activation and antibody response (12, 17). Taking all these data into consideration, we suggest that cross-linking of CD19 with a high affinity mAb leads to the impairment of its functional activity, explaining why the functional outcome of LY3541860 binding on human B cells resembles CD19 deficiency. This observation indicates that, to perform its function, CD19 must be able to move freely on the surface of the B cell and that high-affinity binding of LY3541860 interferes with this process, leading to the inhibition of B cell function.

Alternatively, it is possible that binding of LY354860 leads to internalization of CD19, therefore reducing the numbers of surface CD19 molecules, which may in turn lead to the inhibition of B cell activation. We have observed a significant degree of internalization of LY3541860 in B cells upon binding (Supplemental Figure 8). However, we were unable to assess the reduction of surface CD19 upon binding of LY3541860 due to the fact that it interferes with binding with tested commercial anti-CD19 antibodies. Therefore, internalization of CD19 can contribute the mechanism of action of LY354860, but it needs to be further explored in future studies.

Some other B cell–inhibiting nondepleting antibodies have been described in the literature — for example, anti-CD79 targeting antibody (40), which inhibits B cell response via induction of B cell anergy. We have considered that induction of anergy could explain the mechanism of action of LY3541860. Several experiments were conducted in order to explore this hypothesis; however, we were unable to demonstrate the reduction of calcium flux or downregulation of surface IgM and IgD expression upon LY3541860 binding (Supplemental Figure 9). Therefore, we concluded that inhibitory activity of LY3541860 is unlikely to be associated with the induction of B cell anergy.

Since LY3541860 was developed as a drug candidate for the treatment of autoimmune disorders, it is important to understand whether this new drug candidate possesses any properties to be superior to the already-existing B cell–targeting drugs. We hypothesize that targeting of CD19 with a nondepleting antibody can provide additional efficacy due to the effect on CD19^+^CD20^–^ B cell subsets (plasmablasts and PCs) and advantages of a mechanism of action that does not depend on ADCC and that might be defective in the context of autoimmune inflamed tissues (5). 

In summary, we propose LY3541860 as a B cell–inhibiting nondepleting drug candidate. LY3541860 has potential to demonstrate superior efficacy compared with approved B cell–targeting therapies without causing long-lasting B cell depletion, suggesting highly desirable reversible B cell inhibition.

## Methods

### Phage panning and antibody optimization.

The CD19 antibody C323 was isolated from a fully human antibody phage display library using a cell-based panning approach against human embryonic kidney cells (HEK-293) cotransfected with human CD19 and human CD21 (41, 42). A negative pan against the parental HEK-293 cells was performed to remove nonspecific cell binders.

Engineering of C323 was carried out using Kunkel mutagenesis as described previously (43). NNK degenerate codon libraries (N = A/C/G/T, K = G/T, NNK degenerate codon represents 32 possible codons coding for all 20 amino acids) of all 6 CDRs were screened for affinity maturation, and targeted VVK degenerate codons libraries (V = A/C/G, VVK degenerate codon represents 18 possible codons coding for 12 amino acids excluding Trp, Leu, Ile, Met, Val, Phe, Tyr, and Cys) were screened at select hydrophobic positions. Libraries were screened by capture lift with biotinylated CD19 ECD. Identified mutations were sequenced, expressed as periplasmic Fab in *E*. *coli*, and analyzed by ELISA.

Antibodies or Fabs were captured on ELISA plates coated with either goat anti–human Fc antibody (Southern Biotech, 2046-01) or goat anti-human κ antibody (Southern Biotech, 2060-01). A titration of biotinylated CD19 was added to the plates, and the captured biotinylated antigen was detected by alkaline phosphatase–conjugated neutravidin (Thermo Fisher Scientific).

### Surrogate CD19 antibody generation.

The rat anti-mCD19 antibody 1D3 sequence was cloned from mRNA isolated from the ATCC hybridoma HB-305 using RNeasy Micro and OneStep RT-PCR kids (Qiagen). The CDRs were grafted into the mouse IGHV1S12*01 and IGKV170 germline frameworks with mouse IgG1/κ constant sequences. This murinized 1D3 clone was then affinity matured as described above to generate the surrogate CD19 antibody.

### Expression and purification.

Antibodies and Fab were expressed in CHO cells (44). Capture from cell culture media was accomplished by affinity chromatography using either MabSelect SuRE (Cytiva Life Sciences) for antibodies or CaptureSelect KappaXL (Thermo Fisher Scientific) for Fab. Subsequent purification was done by size exclusion chromatography using Superdex 200 for antibodies and Superdex 75 for Fab (Cytiva Life Sciences).

Human CD19 ECD (amino acids 21–289) was fused to the N-terminus of C-terminally His-tagged human serum albumin (HSA) and expressed in the FreeStyle 293-F system (Thermo Fisher Scientific). Protein was captured from cell culture media by HisPur Ni-NTA (Thermo Fisher Scientific) and further purified by Superdex 200 (Cytiva Life Sciences). Purified CD19-HSA-His was biotinylated with EZ-Link Sulfo-NHS-LC biotin (Thermo Fisher Scientific).

### Hydrophobic interaction chromatography.

Analytical hydrophobic interaction chromatography (HIC) was performed using a 4.6 mm ***×*** 3.5 cm Butyl-NPR column (Tosoh Bioscience) on an Agilent 1260 HPLC. Separation was achieved using a 25-minute linear gradient from 1.5M ammonium sulfate in 25 mM potassium phosphate (pH 7) to 20% isopropanol in 2 5mM potassium phosphate (pH 7). Approximately 50 μg of antibody was injected, and detection was performed via UV absorbance at 214 nm.

### Binding affinity and kinetics.

Biacore T200 instrument (Cytiva), sensor chip, reagents, and Biacore T200 Evaluation Software Version 3.1 were used for SPR analysis of LY3541860 Fab fragment binding to CD19. The experiment was run at 37°C using 1***×*** HBS-EP+ running buffer (Teknova). Fab was diluted to 1,000 nM in running buffer; it was then 5***×*** serially diluted for a total of 5 concentrations. CD19-Fc fusions (R&D Systems) were captured on the Protein A chip, and Fab dilutions were flowed over the chip for 300 seconds followed by a 900-second dissociation period at a flow rate of 50 μL/min. The chip surface was regenerated with glycine (pH 1.5) between cycles. Double-referenced data were fit using the 1:1 binding model.

The equilibrium binding affinity of LY3541860 to cell-bound CD19 was measured by SET at 37°C using CHO cells stably expressing CD19. Prior to analysis, cells were fixed in 1% paraformaldehyde in PBS to prevent internalization and cell death. Cell titration series (~11 ***×*** 10^6^ diluted down to ~450 cells/mL for human CD19 and ~100 ***×*** 10^6^ diluted down to ~4,200 cells/mL for cynomolgus monkey CD19, both as 2.5***×*** serial dilution series) were prepared in 1% Blocker A (Meso Scale Discovery) in 96-well V-bottom plates with fixed antibody concentrations (200, 40, 8, and 1.6 pM or 5,000, 1,000, 200, and 40 pM for the human and cynomolgus monkey CD19 binding, respectively). Plates were incubated with shaking for 2–4 days to allow binding to reach equilibrium. Following incubation, cells were pelleted by centrifugation (1,000*g*, 10 minutes, room temperature), and supernatant was removed to measure the unbound antibody by MSD. Briefly, a multiarray plate (Meso Scale Discovery) was coated with a goat anti–human Fc antibody (Jackson ImmunoResearch, 109-005-098), LY3541860 was captured, biotinylated goat anti–human Ig primary antibody (Southern Biotech, 2010-08) was added, and then bound LY3541860 was detected with SULFO-TAG streptavidin secondary (Meso Scale Discovery). The K_D_ and a least-common multiplier (LCM) to account for unknown antigen concentrations on cells were globally fit from the raw MSD data for the 4 SET series to an equilibrium-binding equation (45) using nonlinear regression in GraphPad Prism.

### ADCC and CDC assay.

For the ADCC assay, the Wil2-s cell line expressing CD19 and CD20 were used as target cells, and Jurkat cell lines expressing functional FcγRIIIa (V158)-NFAT-Luc were used as the effector cell line. The target cells were plated and incubated with 50 μL of varying concentrations of rituximab or LY3541860 for 1 hour at 37°C. After incubation, Jurkat FcγRIIIa (V158)-NFAT-Luc were then plated, resulting in 1:3 target/effector ratio and incubated for 4 hours at 37°C. After incubation, One-glo Ex (Promega) was added to the plate, and luminescence was read using a BioTek

For CDC assay, cells from the Wil2-s cell line expressing CD19 and CD20 were used as target cells. The target cells were then plated and incubated with 50 μL of varying concentrations of rituximab or LY3541860 for 1 hour at 37°C. After incubation, complement from human serum (Quidel) was diluted in assay media 1:6. Diluted complement was added to the assay plate in 50 μL aliquots and incubated for 2 hours at 37°C. After incubation, 100 μL Cell Titer Glo (Promega) was added to each plate, and luminescence was read using a BioTek.

### PBMC and primary B cell isolation.

Leukocyte Reduction System–WBC (LRS-WBC) samples from healthy volunteers were obtained from San Diego Blood Bank (San Diego, California, USA). Peripheral blood mononuclear cells (PBMCs) were separated by Ficoll-paque plus (GE Healthcare) density gradient centrifugation (1,000*g*, 10 minutes, room temperature). PBMCs were harvested, and primary human B cells were isolated by negative selection using human B cell enrichment kit (StemCell Technologies) following the manufacturer’s instructions. The purity (>95%) of mature primary B cells (CD20^+^, CD19^+^, and CD3^–^ populations) was determined by flow cytometry.

### B cell apoptosis assay.

Human primary B cells were plated at 1 ***×*** 10^6^cells/mL. Cells were treated with indicated concentrations of LY3541860 or isotype control for 24 hours at 37°C and 5% CO_2_. B cell apoptosis was measured by flow cytometry using annexin V staining (Invitrogen) in combination with fixable viability dye. Cells were stained with the appropriate combination of fluorochrome-conjugated antibodies for 30 minutes at 4°C to identify B cell surface markers: CD19 APC (clone SJ25CI, BioLegend), CD20 PerCP-Cy5.5 (clone 2H7, BD Pharmingen), annexin V (Invitrogen), and fixable viability dye eFluor 780 (eBioscience, 65-0865). Apoptotic B cells were defined as live/annexin V^+^/CD20^+^. Samples were acquired on a BD Fortessa X-20 and results were analyzed using FlowJo software.

### Binding specificity and potency of LY3541860 in human whole blood.

LY3541860 and isotype control antibodies were directly conjugated to Alexa Fluor dye using AF647 labeling kit (Thermo Fisher Scientific, A20186) following manufacture protocol. LY3541860-AF647 and isotype control–AF647 antibodies were serial diluted. EDTA-treated human whole blood was treated with LY3541860-AF647 or isotype control–AF647 antibodies and appropriate combinations of fluorochrome-conjugated antibodies (CD3 BV605 [BioLegend, clone OKT3], CD20 PerCP Cy5.5 [BD Pharmingen, clone 2H7], CD45 BV421 [BioLegend, clone HI30], and viability dye [65-0865, eBioscience]) for 30 minutes at 4°C in the dark. Fluorescence minus one (FMO) controls were prepared for each antibody and used as gating controls for staining panel. After 30 minutes of incubation, RBCs were lysed and washed in FACS buffer. Cells were then acquired on the BD Fortessa X-20. Dead cells were excluded by viability dye, and at least 25,000 to 50,000 events gated on living cells were analyzed for each sample; results were analyzed using FlowJo software.

### B cell proliferation.

Human primary B cells were cultured in 96-well plates, and cells were pretreated with various concentrations of LY3541860 or isotype control antibodies as indicated for 30 minutes. B cell proliferation was induced by 2 μg/mL anti–human IgM (9022-01, Southern Biotech) plus 12 μg/mL rabbit anti–mouse IgG (31190, Thermo Fisher Scientific) for 2 days at 37°C and 5% CO_2_. Cells were then pulsed with [3H]-thymidine (PerkinElmer) for 18 hours of cell culture. [3H]-thymidine incorporation was measured by 2450 Microplate Counter and expressed as a cell count per minute (CCPM).

Alternatively, cell proliferation was assessed using CellTiter-Glow Luminescent Cell Viability Assay (Promega) as follows. On day 3 of culture, 100 μL of cells was transferred into opaque-well, 96-flat bottom plates. In total, 100 μL of medium alone was transferred to wells designated for background luminescence. A total of 100 μL of CellTiter-Glow Reagent was added to cell culture medium present in each well and placed on an orbital shaker for 2 minutes to induce cell lysis. Plates were covered and incubated for 15 minutes to stabilize the luminescent signal. The signal was recorded using Cytation5 imaging reader.

### CpG-induced B cell activation in human whole blood.

In total, 100 μL of EDTA-treated human whole blood was plated and pretreated with various concentrations of LY3541860 or isotype control antibodies for 30 minutes at 37°C. For induction of CD69 expression, 2.5 μg/mL of CpG (InVivogen) as final concentration was added in complete medium. No stimulation was designated as baseline. The plates were kept at 37°C and 5% CO_2_ overnight. Then, cells were stained with the following antibodies for 30 minutes at 4°C: CD69 BV605 (clone FN50, BioLegend), CD20 PerCP Cy5.5 (clone 2H7, BD Pharmingen), CD19 APC (clone SJ25CI, BioLegend), and viability dye (eBioscience, 65-0865). FMO controls were used as gating controls. Cells were washed with staining buffer and run on the BD Fortessa X-20. Dead cells were excluded by viability dye, and at least 25,000–50,000 live cells were analyzed for each sample; results were analyzed using FlowJo software.

### Memory B cell differentiation into plasmablasts.

Memory human B cells were isolated from healthy donor PBMCs using memory B cell isolation kit (Miltenyi Biotec, 130-093-546). Human primary memory B cells were resuspended at 1 ***×*** 10^6^ cells/mL and cultured at 37°C. Cells were pretreated with varying concentrations of either isotype control or LY3541860 as indicated for 30 minutes and stimulated with 50 ng/mL anti-CD40, 200 ng/mL BAFF, 1 ng/mL IL-2, and 100 ng/mL IL-21 (all from R&D Systems) for 5 days. Cells were washed and stained with the following combination of fluorochrome-conjugated antibodies — CD38 PE (clone HB-7), CD3 FITC (clone HIT3a), CD19 APC (clone SJ25CI, all from BioLegend), CD20 PerCP-Cy5.5 (clone 2H7, BD Pharmingen), and fixable viability dye eFluor 780 (65-0865, eBioscience) — in staining buffer for 30 minutes at 4°C to identify differentiation of memory B cells into plasmablasts. Samples were acquired on a BD Fortessa X-20, and results were analyzed using FlowJo software. Plasmablasts were defined as CD38^bright^CD20^lo^ B cells.

### Internalization assay.

A labeled F(ab’)2 targeting human Ig Fcγ fragment (F[ab’]2-TAMRA-QSY7) was used as a probe to track internalization. The test antibody was incubated with probe at 4°C for 30 minutes to form the complex and was added to the isolated human B cells. The final concentration of the test antibody was 2 μg/mL. Cells were incubated for 24 hours at 37°C in a CO_2_ incubator. Cells were then washed twice with 2% FBS PBS and resuspeneded in 2% FBS PBS with a viability dye (SYTOX Green, Invitrogen). Data were collected on a BD Fortessa X-20 and analyzed in FlowJo.

### In vivo B cell activation in humanized NSG mice.

Female NSG mice (NOD.Cg-Prkdcscid Il2rgtm1Wjl/SzJ, JAX Labs, stock no. 05557) were housed 3/cage at 72°F under a 12-hour light/dark cycle and allowed food and water ad libitum (*n* = 33). Human PBMCs were isolated from LRS tubes obtained from the San Diego Blood Bank using SepMate 50 Ficol preparation tubes according to the manufacturer’s instructions (StemCell Technologies). Freshly isolated PBMCs were suspended in PBS at 1.2 ***×*** 10^8^ cells/mL, and mice were engrafted with 100 μL PBMC suspension i.v. on day 0 (1.2 ***×*** 10^7^/mouse, *n* = 29). Four mice were not administered PBMCs as nonengrafted controls. On day 1, mice were divided into 3 weight-matched groups and dosed with isotype control or LY3541860 at 0.01 or 1.0 mg/kg s.c. (200 μL/mouse, *n* = 10, 10, and 9, respectively). Dosing continued once weekly for the remainder of the experiment. Blood was collected by tail snip into heparin-coated capillary tubes on days 6 and 10. Spleens were harvested and processed to single-cell suspensions for FACS analyses.

### HNSG mice plasma analysis.

Blood from the cardiac puncture was collected into EDTA-coated tubes and clarified by centrifugation (3,000*g*, 10 minutes, room temperature), and the resultant plasma was stored at –80°C for future processing. Plasma IgM levels were measured using the Mesoscale Discovery human isotyping panel (stock no, K15203D-4) according to manufacturer instructions.

### FACS analysis of hNSG splenocytes.

Single-cell suspensions of mouse spleens were used for FACS analysis. Cells were plated in 96-well plates and stained with the appropriate combination of fluorochrome-conjugated antibodies for 30 minutes at 4°C to identify B cell activation markers: hCD45-BV421 (clone HI30), CD86 BV650 (clone IT2.2), CD3 APC (clone OKT3), CD19 FTIC (clone HIB19, all from BioLegend), CD20 PerCP-Cy5.5 (clone 2H7, BD Pharmingen), and fixable viability dye eFluor™ 780 (65-0865, eBioscience). At least 250,000 events gated on living cells were analyzed for each sample. Samples were acquired on a BD Fortessa X-20, and results were analyzed using FlowJo software.

### In vivo efficacy studies in CIA, NOD, and PLP-induced EAE mouse models.

For CIA model, 6- to 7-week-old male DBA/1 mice (Envigo) were immunized with type II collagen in FCA on day 0, animals were randomized into treatment groups on day19, and treatment was initiated. Clinical scores of all 4 paws were recorded daily. On day 21, mice were administrated a boost injection of type II collagen in FCA. On day 42, mice were euthanized and serum was collected for anti-collagen ELISA. The right forepaw, right hindpaw, and right knee were collected in 10% neutral buffered formalin for histopathological analysis. For histology, the paws were demineralized, embedded in paraffin, sectioned, and stained with toluidine blue at Bolder BioPath. All slides were examined microscopically by a pathologist at Bolder BioPath. Serum samples were analyzed using the mouse anti–bovine type II collagen IgG antibody assay kit (Chondrex Inc.) per manufacturer instructions. Serum samples were diluted 1:40,000 except for the nondiseased group samples, which were diluted 1:1,000.

For NOD model, 8-week-old female NOD/ShiLTJ mice (The Jackson Laboratory) were used. After acclimation, mice had blood glucose levels measured weekly using an Accu Check Aviva blood glucose meter (Roche, 06870287001) and test strips (Roche, 06908373001, lot 497064) with blood samples collected from the tail. Any blood glucose value that was too high to read was entered as 600 mg/dL. Any value above 240 mg/dL was considered an indication of diabetes (46). If a mouse had 2 consecutive weeks with readings of 600 mg/dL, it was removed from study and euthanized.

For EAE study, 9- to 10-week-old SJL female mice (Taconic) were used. Mice were immunized by shallow s.c. injection in each flank with PLP139-151/CFA and 75 ng pertussis toxin (PTX) via i.p. injection on the same day. Animals were randomized based on the body weight into study groups with 15 mice in each group. Treatment started on day 8, and EAE score was collected daily with scale 0–6 in 0.5 unit increments. All the mice were sacrificed on day 42.

### Anti-CD40 B cell activation in vivo.

Six-week-old C57BL/6 mice (The Jackson Laboratory) were injected i.v. with 500 μg/kg of anti-mCD19 or isotype control antibody. Mice were given 1.5 mg/kg of anti-CD40 (clone 1C10, eBioscience, 16-0401-86) via retroorbital vein either 1 hour after the i.v. injection (before washout) or 4 weeks after the i.v. injection (after washout). Mice were euthanized 24 hours after anti-CD40 injections; splenocytes were analyzed by FACS.

### Immunofluorescence staining and microscopy.

Human primary B cells were plated at 5 ***×*** 10^6^cells/mL in 6-well plates. Cells were treated with indicated concentrations of LY3541860 conjugated to Alexa Fluor 647 or isotype control conjugated to Alexa Fluor 647 for 30 minutes or overnight at 37°C and 5% CO_2_. Upon treatment, B cells were stimulated overnight.

The following day, cells were washed with PBS and fixed in 4% PFA at room temperature. After washing with PBS, cell pellets were resuspended in 20–50 μL of PBS. Primary human B cells were then smeared on poly-L-lysine–coated coverslips. Upon drying, cells were washed again and permeabilized with 0.3% PBS-Triton. Cells were blocked with normal goat serum and stained with goat anti–human IgM-Cy3 (50-194-1656, Jackson ImmunoResearch) and LY3541860 conjugated to Alexa Fluor 647, at 4°C overnight. The following day, cells were washed and mounted on glass slides using VECTASHIELD Vibrance Antifade Mounting Medium with DAPI (Vector Laboratories, H-1800-10). Primary human B cells were imaged using the Keyence BZ-X800 fluorescence microscope ***×***60 objective and analyzed using ImageJ (NIH) software. For all immunofluorescence experiments, B cells from 5 donors total and at least 5 randomly chosen regions per condition and donor were imaged and analyzed. ImageJ was used to count total number of B cells based on nuclear staining, while capping events were counted manually in a blinded fashion.

### Phospho-flow cytometry.

Human B cells were isolated as described above and plated at 1***×*** 10^6^ cells/mL in complete RPMI. LY3541860 or isotype control antibody was added at 1 μg/mL, and cells were incubated in the presence of the antibody overnight at 37°C and 5% CO_2_, after which cells were stimulated with 2 μg/mL anti–human IgM (Southern Biotech, 9022-01) plus 12 μg/mL rabbit anti–mouse IgG (Thermo Fisher Scientific, 31190) for 10 minutes, followed by addition of 1***×*** Lyse/Fix buffer (BD Bioscience) for 5 minutes. Cells were washed, fixed in ice-cold methanol (MilliporeSigma) on ice for 20 minutes, washed in FACS buffer, and stained with anti–pERK1/2-PE (clone MILANBR, Invitrogen) and anti–pAKT (S473)-APC (clone SDRNA, Invitrogen) antibodies for 1 hour at room temperature. Next, cells were washed twice in FACS buffer, acquired on a BD Fortessa X-20, and results were analyzed using FlowJo software.

### Cynomolgus monkey treatment with LY3541860, blood collection, and analysis.

The cynomolgus monkey study was conducted at LabCorp, according to their standard operating procedures. Young adult to adult male and female naive monkeys weighing 2–5 kg were administered LY3541860 as single i.v. or s.c. injection. Blood was collected over 672 hours after dose and processed to plasma to determine LY3541860 concentrations. The plasma exposure of LY3541860 was measured by ELISA using CD19 antigen, CD19-Fc (R&D Systems, 9269-CD), as the capture reagent. Standards, control, and samples were detected with mouse anti–human IgG4-pFc’ horseradish peroxidase (HRP; Southern Biotech, 9190-05). The standard curve ranged from 0.78 to 50 ng/mL.

For flow cytometry analysis, FACSLysed fixed and frozen whole blood samples were used to identify target occupancy by LY3541860 on B cells, as well as frequency of B cells (CD45^+^CD20^+^) in cynomolgus monkey whole blood. The following fluorescently labeled antibodies were used: anti–NHP CD45-FITC (BD Bioscience, 557803), anti–huCD20 PerCP-Cy5.5 (BD Bioscience, 560736), and anti-huCD19 APC (Beckman Coulter, IM2470U). Target engagement was measured by analyzing changes in the amount of unoccupied CD19 in CD45^+^CD20^+^ B cells. Percent unoccupied CD19 was then calculated by subtracting the 100% occupancy mean fluorescence intensity (MFI) from both the predose and a given time point for an animal’s samples, and then dividing the resultant time point MFI by the predose MFI.

### Nanostring analysis of gene expression.

mRNA expression levels were determined using the NanoString platform and the “Human Autoimmune Profiling” reporter panel, following manufacturer-recommended methods. RNA from at least 1 ***×*** 10^6^ B cells cultured in vitro were isolated using the RNEasy Mini Kit (Qiagen, 74104). RNA was diluted to 20 ng/μL in RNase free water, and a total of 100 ng (in 5 μL) was used for probe hybridization. Raw mRNA counts were normalized using the nSolver Advanced Analysis software (version 2.0.115) following manufacturer-recommended data processing methods. In brief, raw mRNA counts were scaled to intrasample binding controls and were then normalized to housekeeping genes following variance-minimizing housekeeping gene selection within the nSolver analysis software. Results are reported as log_2_ expression relative to the mean expression of the “No-Treatment” group for each gene. Genes were determined to be differentially expressed if they had an absolute fold change greater than 1.5 and a *P* value less than 0.05 (*n* = 6 for all treatments). Statistical analysis was performed within the nSolver software.

### Statistics.

Data were graphed and statistics calculated using Prism Software (GraphPad). Differences between groups were determined by either 2-tailed Student’s *t* test or 1-way ANOVA with Dunnett’s or Tukey’s post hoc test and considered significant if *P* < 0.05.

### Animal study approval.

All animal studies were performed in accordance with American Association for Laboratory Animal Care International standards as set forth by the *Guide for the Care and Use of Laboratory Animals* (National Academies Press, 2011). All Lilly-internal in vivo experimental protocols were approved by the Eli Lilly and Company Animal Care and Use Committee.

## Author contributions

JSB, DS, JP, SP, AV, JN, BB, EMS, KK, ERH, CL, JL, JB, RS, JAM, CMW, GLD, and KR designed, performed, and analyzed the experiments. JSB, SP, AV, JP, WYC, YLM, MGC, JAM, and KR wrote the manuscript. MDL, SN, DRW, and AB contributed to the experimental ideas and reviewed the manuscript. EM designed, performed and analyzed experiments.

## Supplementary Material

Supplemental data

## Figures and Tables

**Table 1:**
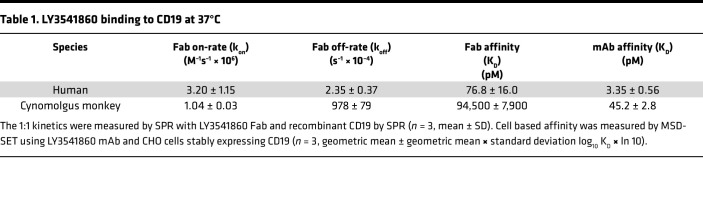
LY3541860 binding to CD19 at 37°C

**Table 2:**
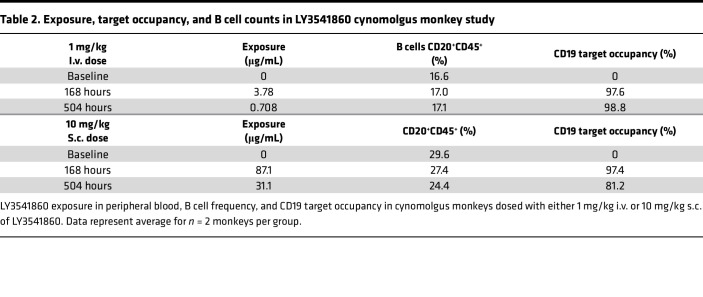
Exposure, target occupancy, and B cell counts in LY3541860 cynomolgus monkey study

## Version 1 07/10/2023

Electronic publication
