# Supplementary material for: A nondepleting anti-CD19 antibody impairs B cell function and inhibits autoimmune diseases
Source: JCI Insight. 2023 Jul 10;8(13):e166137. doi: 10.1172/jci.insight.166137 (PMC10371335; doi:10.1172/jci.insight.166137)
Supplement: Supplemental data [file jciinsight-8-166137-s184.pdf]

Supplemental Materials

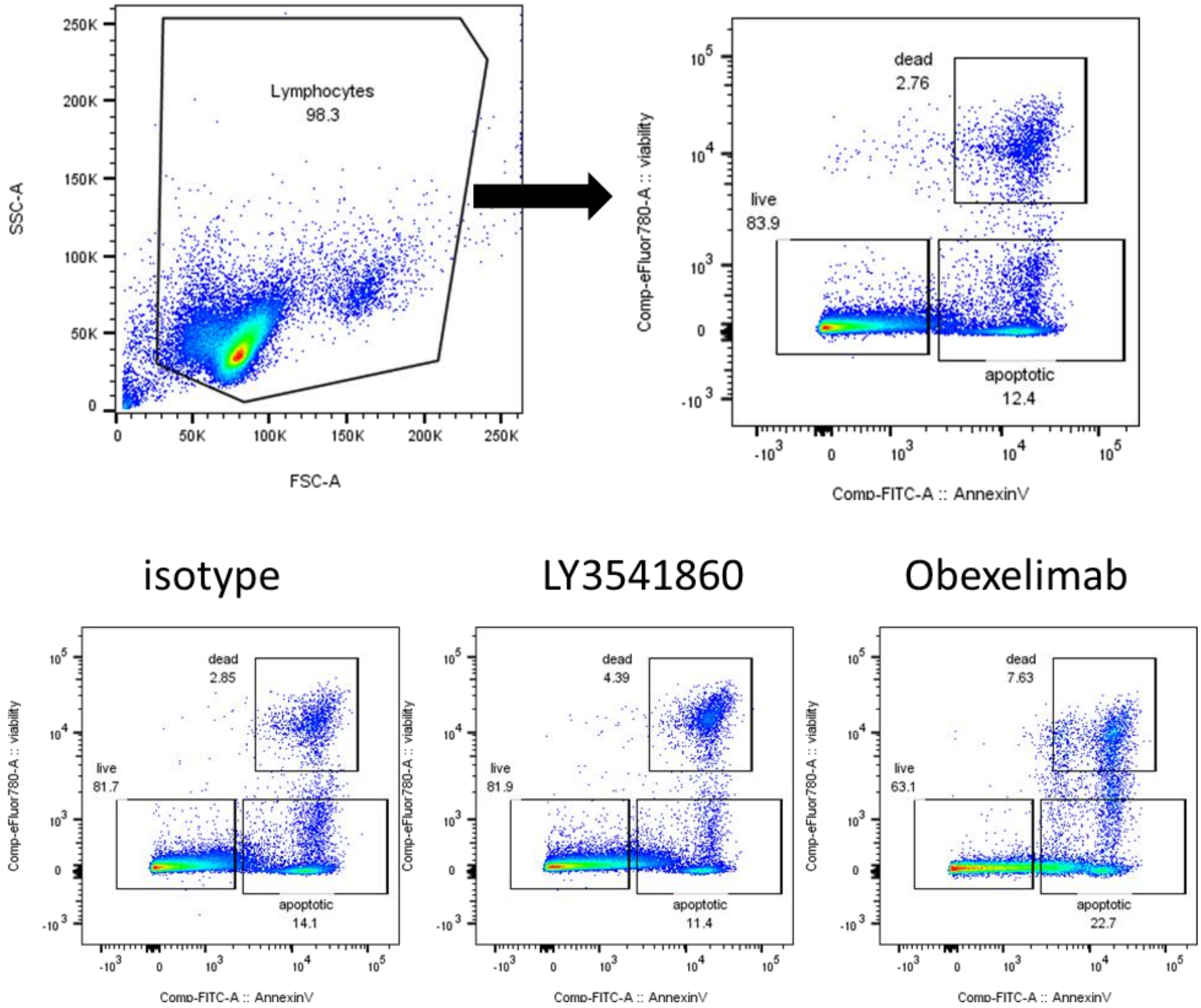

Supplemental Figure 1. Apoptosis assay – gating strategy and representative FACS plots.

Gating strategy (top) and representative FACS plots of B cells treated with 1µg/ml isotype control, LY3541869, or Obexelimab (left to right) for 24 hours.

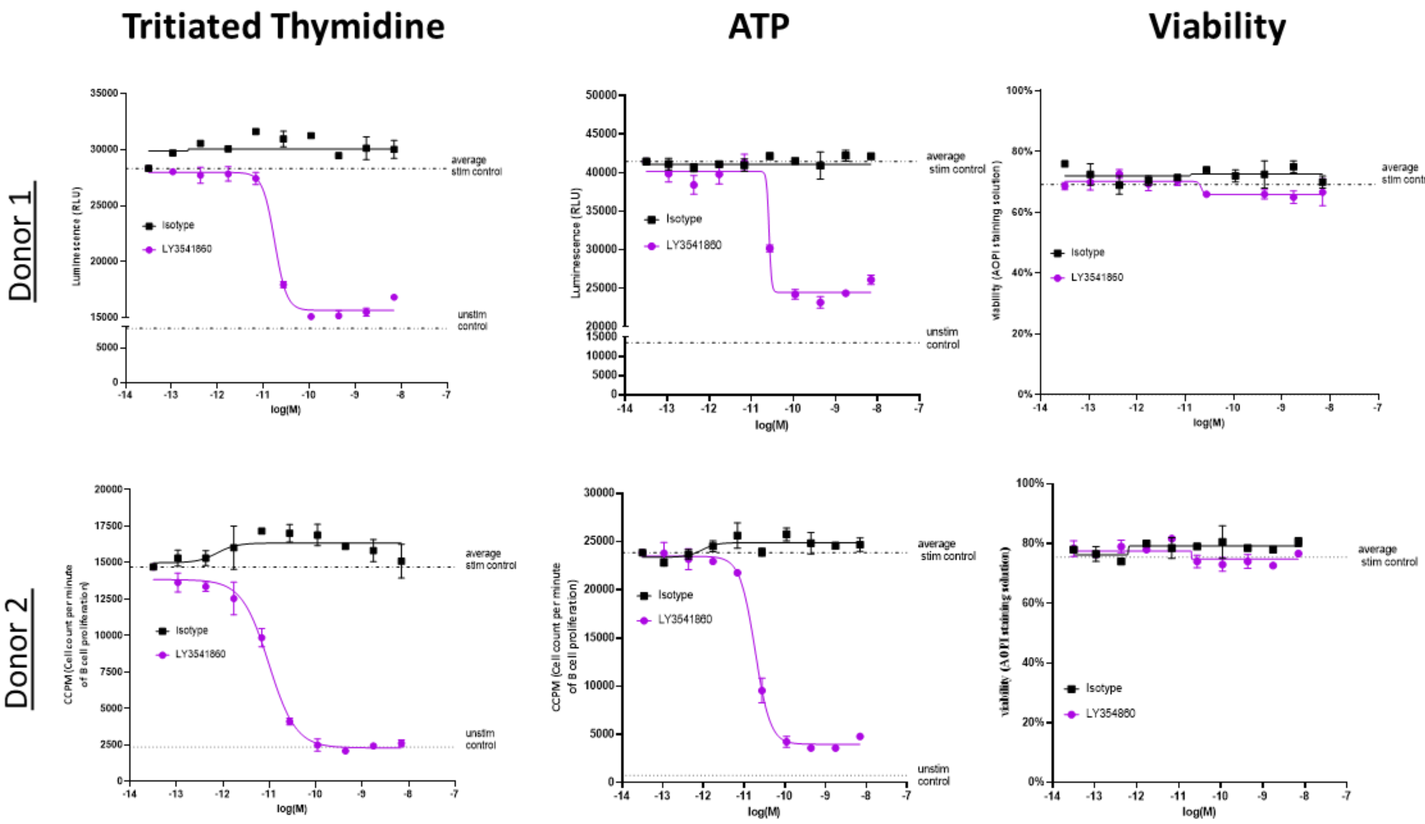

Supplemental Figure 2. Inhibition of B cell proliferation by LY3541860.

Representative data showing a concentration-dependent decrease of B cell proliferation in vitro in two independent donors. Proliferation was detected either using tritiated thymidine (left), or ATP-based assay (middle). Cell viability is demonstrated on the right.

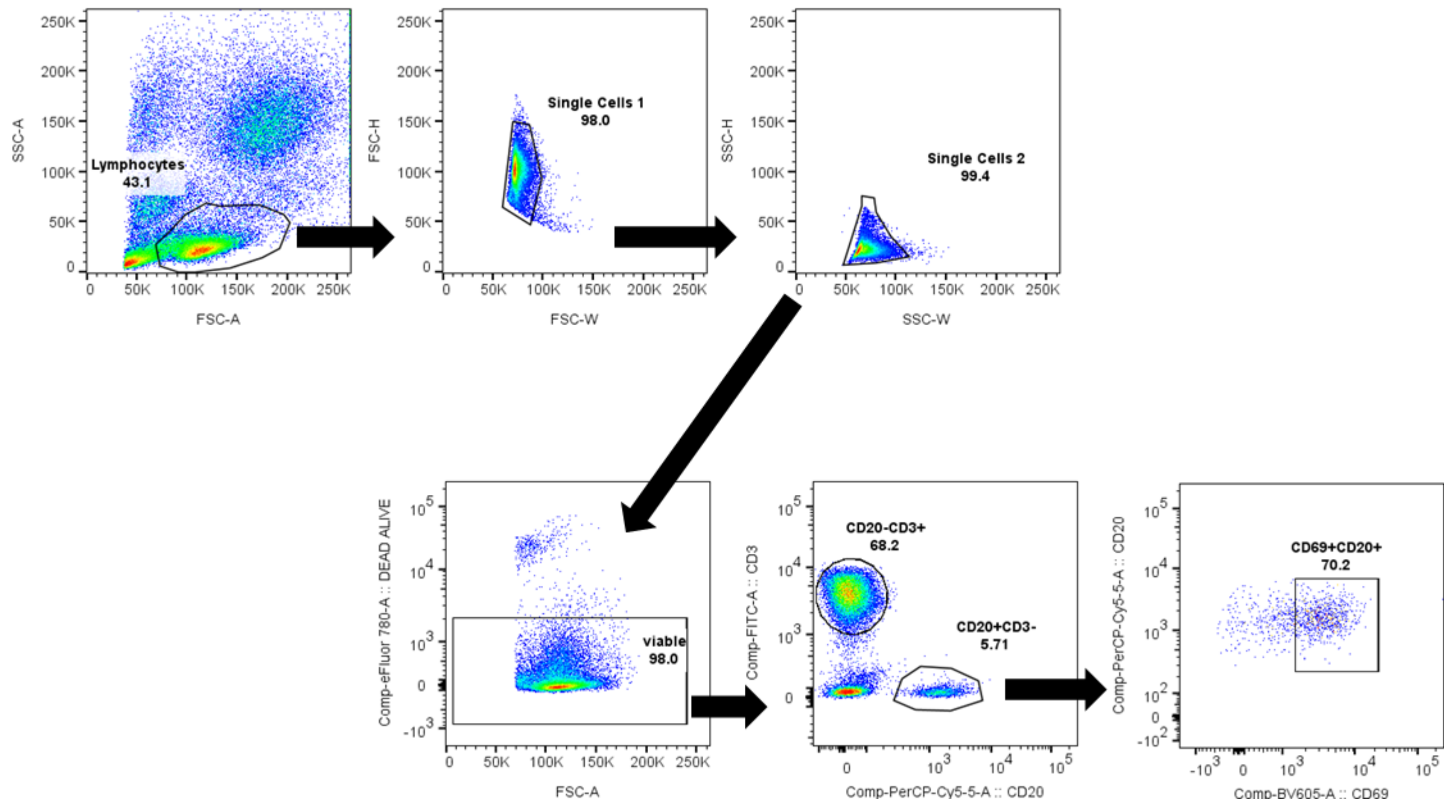

Supplemental Figure 3. Inhibition of CD69 expression – gating strategy

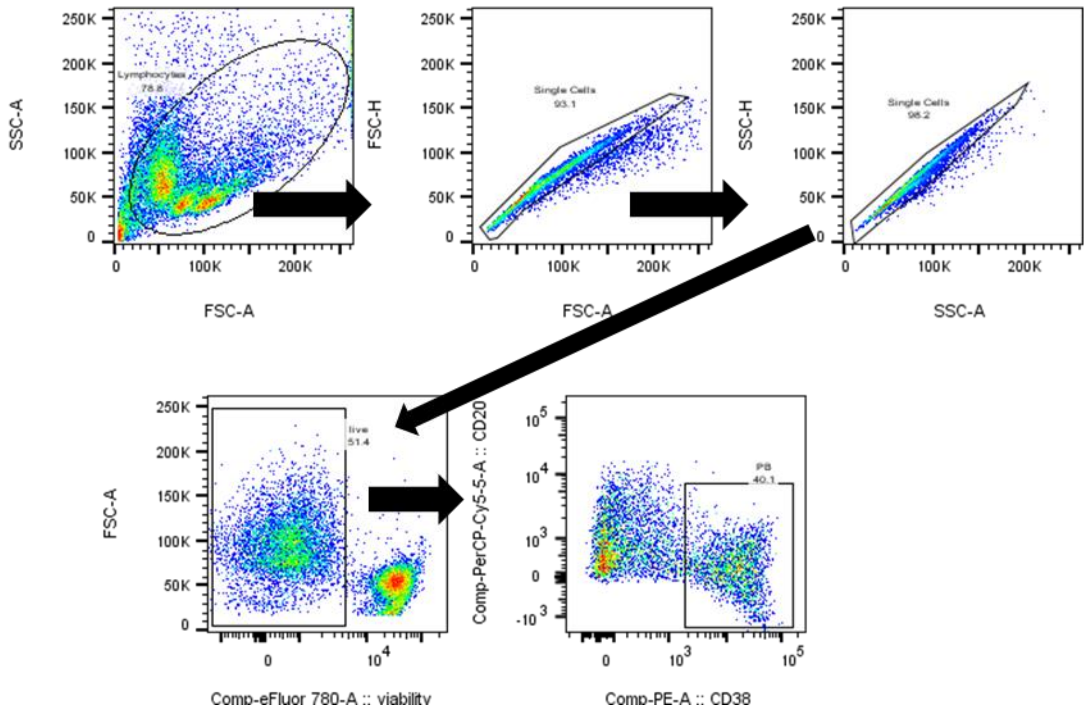

Supplemental Figure 4. Inhibition of plasmablast differentiation – gating strategy

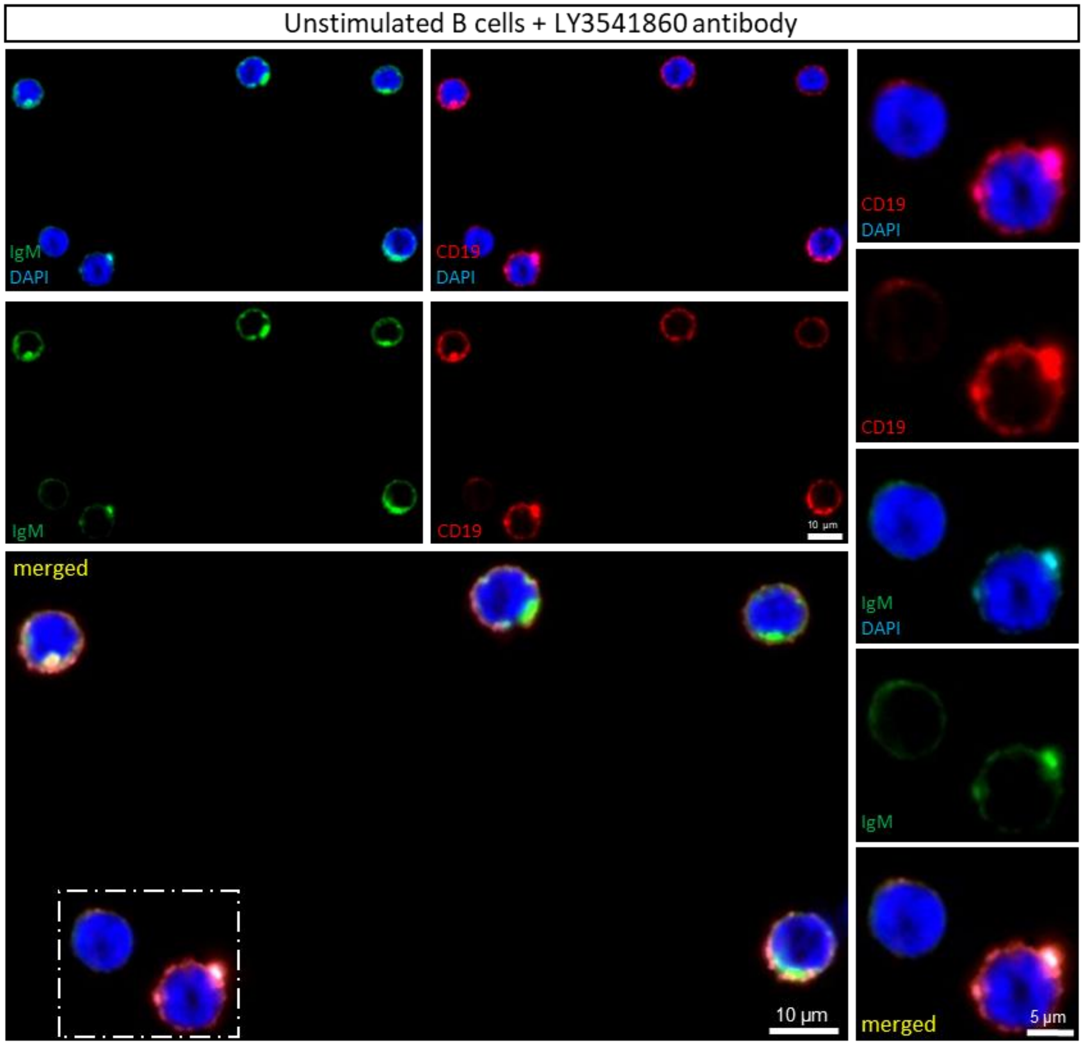

Supplemental Figure 5. LY3541860 doesn't have effect on resting unstimulated B cells

Immunofluorescence images showing primary human B cells after the overnight LY3541860 treatment in the absence of stimulation. CD19 is shown in red, IgM in green and nuclear staining in blue. Dotted line box is shown in higher magnification. Scale bars are 10 µm in lower magnification images including merged image and 5 µm in higher magnification images.

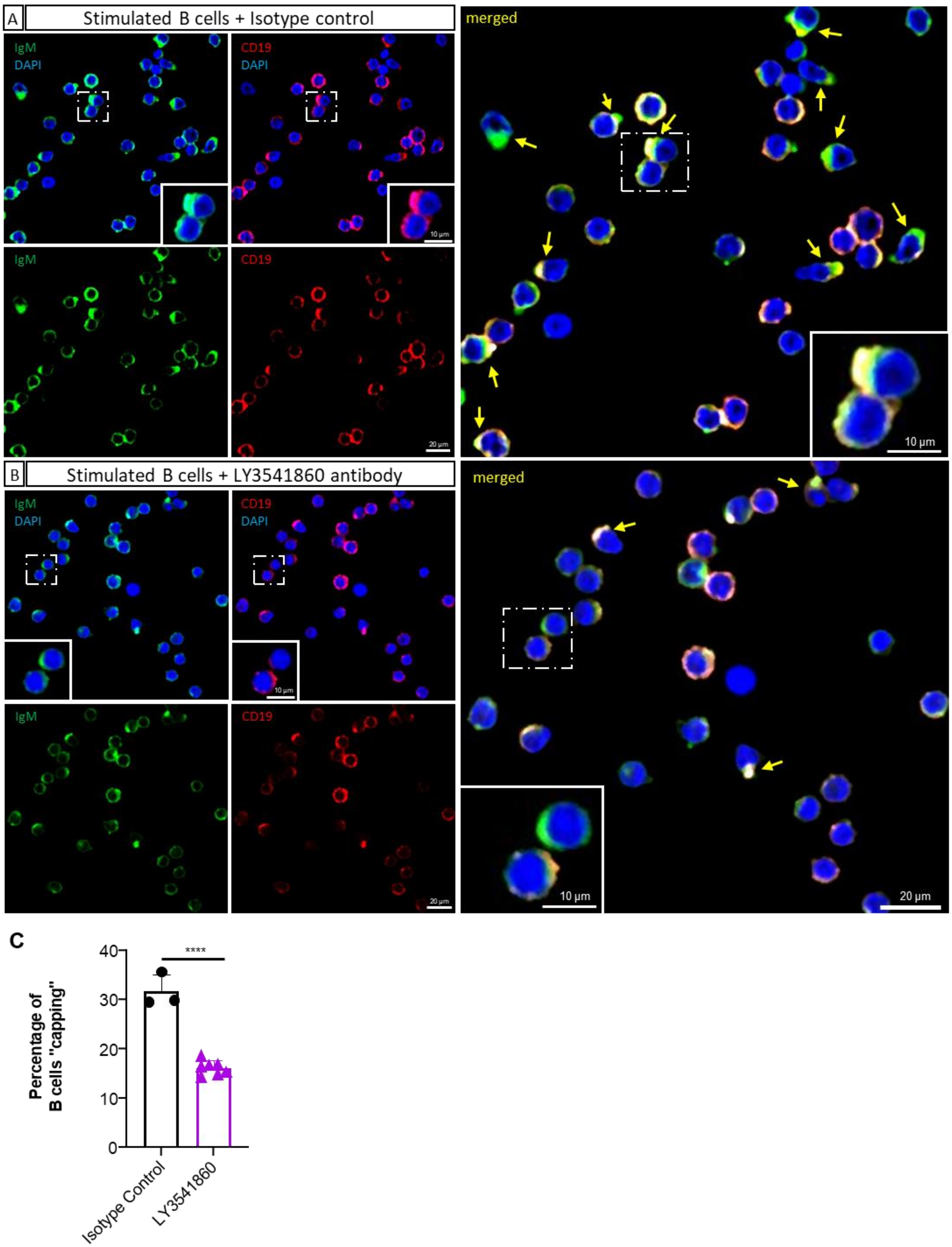
A
Stimulated B cells + Isotype control
IgM
DAPI
CD19
DAPI
10 µm
IgM
CD19
20 µm
merged
10 µm
B
Stimulated B cells + LY3541860 antibody
IgM
DAPI
CD19
DAPI
10 µm
IgM
CD19
20 µm
merged
10 µm
20 µm
C
Percentage of B cells "capping"
****
Isotype Control
LY3541860

Supplemental Figure 6. Inhibition of BCR capping and downstream BCR signaling after 30 minutes of LY3541860 treatment.

A. Immunofluorescence images showing primary human B cells after 30 minutes of Isotype control treatment followed by 24h stimulation. CD19 is shown in red, IgM in green and nuclear staining in blue. Yellow arrows indicate capping. Dotted line box is shown in higher magnification. Scale bars in insets are 10 µm and in the merged image 20 µm. **B.** Immunofluorescence images showing primary human B cells after 30 minutes of LY3541860 treatment followed by 24h stimulation. CD19 is shown in red, IgM in green and nuclear staining in blue. Yellow arrows indicate capping. Dotted line box is shown in higher magnification. Scale bars in insets are 10 µm and in the merged images 20 µm. **C.** Percentage of stimulated B cells "capping" after the LY3541860 or Isotype control treatment. N=3 for isotype control (total of 384 cells) and n=7 for LY354186 (total of 1174 cells) of randomly imaged coverslip regions. *p<0.0001; t-test.

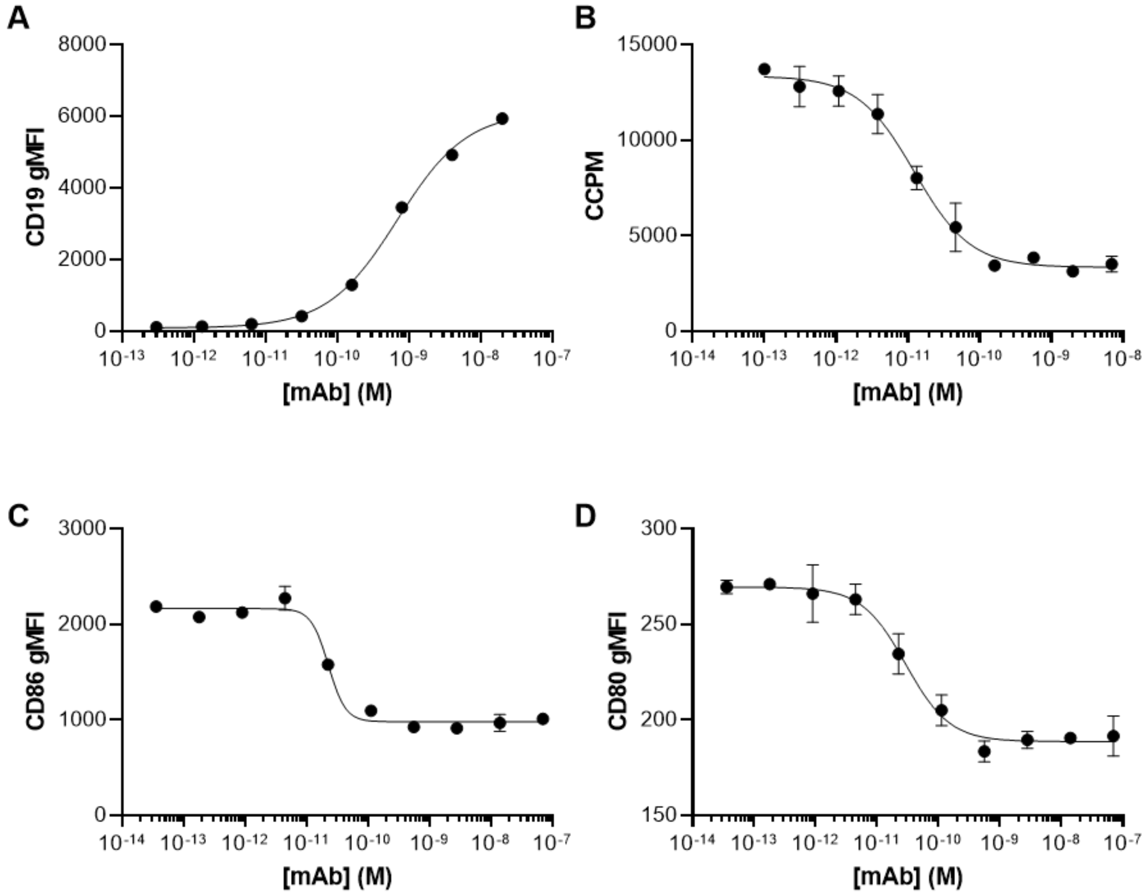

E

	Anti-huCD19 LY3541860	Anti-mCD19
B cell binding affinity	EC50 0.18 nM	EC50 0.7 nM
Potency in inhibition of B cell proliferation	IC50 8 pM	IC50 34 pM
Potency in inhibition of B cell activation	IC50 6 pM	IC50 23 pM

Supplemental Figure 7. Characterization of anti-mCD19 non-depleting antibody.

A.Binding of fluorescently labeled anti-mCD19 antibody to mouse splenic B cells. **B**. Inhibition of (Fab)^{2}anti-mouse IgM induced mouse B cell proliferation by anti-mCD19 antibody. **C, D**. Inhibition of (Fab)^{2}anti-mouse IgM induced CD86 (**C**) and CD80 (**D**) expression on mouse splenic B cells by anti-CD19

antibody. **E**. Comparison of anti-huCD19 (LY3541860) and anti-mCD19 antibody potency in B cell assays.

All assay performed at least two times using B cells obtained from at least 4 independent C57/Bl6 mice.

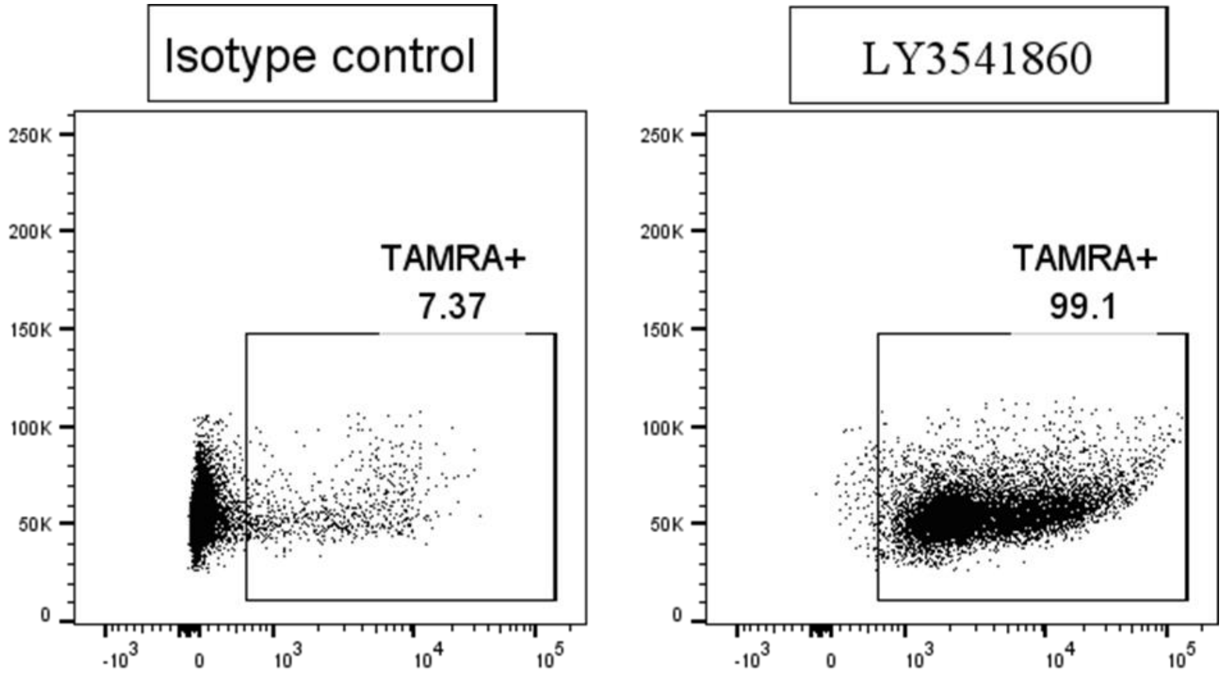

Supplemental Figure 8. Internalization of LY3541860 in primary B cells

Internalization of isotype control (left) or LY3541860 (right) in primary human B cells (detected using TAMRA-labeled F(ab')2 targeting human Ig Fcγ fragment (F(ab')2-TAMRA-QSY7) after 24h of incubation.

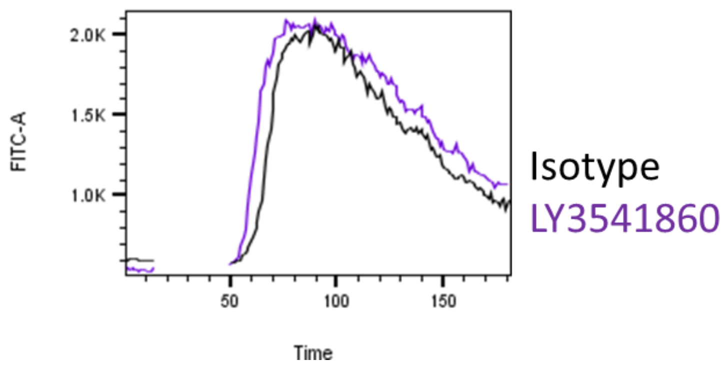

Supplemental Figure 9. LY3541860 doe not affect calcium flux induced by (Fab')2 anti-IgM on primary human B cells.

Table s1. Data represents relative IC50 inhibitory activity of LY3541860 on primary B cell expansion in vitro after 72 hours in culture. IC50 values were generated using a four parameter logistic fit of CCPM as a function of concentration of LY3541860. The average IC50 from 11 independent experiments using a total of 16 different donors was determined to be 0.008 nM ± 0.001 nM

Human Healthy Donors	LY3541860	
	slope	IC_{50} (nM)
Donor 1	-1.70	0.001
Donor 2	-5.80	0.01
Donor 3	-3.80	0.005
Donor 4	-3.00	0.004
Donor 5	-2.30	0.003
Donor 6	-2.80	0.003
Donor 7	-7.70	0.006
Donor 8	-3.00	0.019
Donor 9	-1.60	0.018
Donor 10	-1.70	0.002
Donor 11	-2.00	0.006
Donor 12	-1.80	0.004
Donor 13	-2.00	0.005
Donor 14	-2.00	0.014
Donor 15	-2.20	0.009
Donor 16	-1.20	0.013
Average	-2.79	0.008
SEM		0.001

Table s2. LY3541860 IC_{50} values of CD69 inhibition after CpG stimulation in human whole blood Relative IC_{50} of inhibitory activity of LY3541860 on expression level of CD69 on primary B cells in human whole blood after CpG stimulation, generated using four parameter logistic fit

gMFI as a function of concentration of LY3541860. The average IC_{50} from 4 independent experiments using total of 4 different donors was determined to be 0.006 nM ± 0.003 nM

Donor	IC_{50} (nM)
1	0.008
2	0.001
3	0.001
4	0.013
Average	0.006
